# PINK1-mediated mitophagy supports lung cancer initiation and limits the efficacy of *KRAS*-targeted therapy

**DOI:** 10.1126/sciadv.aef8820

**Published:** 2026-10-16

**Authors:** Diogo Gonçalves, Emma Guilbaud, Léa Di Mascio, Camille Guerrier, Linda Mejri, Sonia Núñez-Vázquez, Lucie Levasseur, Rachel Paul, Corinne Menigot, Christophe Bontoux, Guylène Rignol, Véronique Hofman, Paul Hofman, Marin Truchi, Bernard Mari, Laurent Yvan-Charvet, Johanna Chiche, Sandrine Marchetti, Jean-Ehrland Ricci

**Affiliations:** ^1^ Université Côte d’Azur, INSERM, C3M, Nice, France.; ^2^ Équipe labellisée LIGUE Contre le Cancer, Nice, France.; ^3^ IHURespirERA, FHU OncoAge, Nice, France.; ^4^ Cancer Signaling and Microenvironment Program, Fox Chase Cancer Center, Philadelphia, PA, USA.; ^5^ Department of Pathology, Cancer University Institute of Toulouse-Oncopole, University Hospital of Toulouse, 1 av Irène Joliot-Curie, Toulouse, 31059, France.; ^6^ Université Côte d’Azur, INSERM, CNRS, IRCAN, Nice, France.; ^7^ CHU de Nice, LPCE, Hospital-Integrated Biobank (BB-0033-00025), Nice, France.; ^8^ Université Côte d’Azur, CNRS, IPMC, Sophia-Antipolis, France.

## Abstract

Mitophagy, the selective degradation of mitochondria by lysosomes, is a major mitochondrial quality-control pathway, yet its role in lung cancer initiation and therapeutic response remains poorly understood. We investigated PINK1-dependent mitophagy in human lung adenocarcinoma and directly monitored mitophagy in vivo using mito-QC reporter mice during lung tumor development. We found that PINK1 activity and mitophagy are increased in human tumors and are activated from the earliest stages of tumorigenesis. Genetic inhibition of PINK1 impaired oncogenic transformation but had little impact once tumor cells were established, identifying mitophagy as a critical early adaptation during lung cancer initiation. PINK1 deficiency markedly enhanced the efficacy of *KRAS*-targeted therapy in vitro and in vivo. Together, our findings identify PINK1-dependent mitophagy as an early vulnerability in *KRAS*-driven lung cancer and provide a rationale for targeting mitophagy to improve the efficacy of *KRAS*-targeted therapies.

## INTRODUCTION

Mitochondria occupy a central position in the biology of cancer cells, functioning not only as energy-producing organelles but also as key regulators of cellular fitness under stress (*1*). Beyond their classical role in oxidative phosphorylation, mitochondria integrate metabolic cues, redox fluctuations, and apoptotic signals, thereby shaping how tumor cells respond to hostile microenvironments and anticancer therapies (*2*). These adaptive features rely heavily on mitochondrial quality-control processes, including mitochondrial dynamics and biogenesis, and selective mitochondrial turnover (mitophagy), which collectively safeguard functional organelle networks and enable rapid metabolic reprogramming (*3*, *4*).

Mitophagy, a selective form of macroautophagy, is the principal mechanism through which cells eliminate damaged or dysfunctional mitochondria to preserve metabolic fitness and redox homeostasis (*5*). This process is essential for maintaining mitochondrial quality, and its dysregulation has been implicated in diverse pathological conditions, including neurodegeneration and cancer (*6*). While the importance of mitophagy in Parkinson’s disease is well established, its precise contribution to tumor initiation, maintenance, and therapeutic resistance remains incompletely understood and appears to be highly context dependent (*7*, *8*). Several mitophagy pathways have been described [reviewed in (*9*)], but the serine/threonine kinase PTEN–induced kinase 1 (PINK1)–Parkin axis remains the most extensively characterized mechanism linking mitochondrial damage to selective organelle clearance. In healthy mitochondria, PINK1 is imported across the mitochondrial membranes and cleaved by inner-membrane proteases, resulting in rapid proteasomal degradation (*10*). Mitochondrial depolarization—a central feature of mitochondrial stress—prevents PINK1 cleavage, allowing full-length PINK1 to accumulate and become activated on the outer mitochondrial membrane (OMM). Activated PINK1 phosphorylates several substrates including ubiquitin at Ser^65^ and indirectly RAB8A at Ser^111^ (*11*), which participates in the recruitment and activation the RBR E3 ubiquitin ligase Parkin. Parkin amplifies the ubiquitin signal on OMM proteins, which in turn recruits mitophagy adaptors, ultimately linking damaged mitochondria to the autophagic machinery for lysosomal degradation. Although Parkin-mediated mitophagy is well established in neuronal biology, Parkin expression is silenced or lost in many human cancers, including lung tumors and lung cancer cell lines (*12*). By contrast, PINK1 remains broadly expressed and retains the ability to sense mitochondrial depolarization in cancer cells (*13*). This suggests that PINK1 may cooperate with alternative ubiquitin ligases to initiate mitophagy in tumors lacking Parkin. Consistent with this idea, we previously showed that in lung cancer cells, PINK1 partners with the RBR E3 ligase ARIH1 to promote mitophagy in response to mitochondrial stress, thereby sustaining mitochondrial quality control and contributing to chemoresistance (*14*). These observations highlight a critical knowledge gap at the intersection of mitophagy, cancer initiation, and therapeutic resistance.

Lung cancer, the first cancer killer worldwide, exhibits notable metabolic heterogeneity. While elevated glycolysis is a hallmark of many tumors, subsets of lung cancers rely heavily on mitochondrial oxidative metabolism and use diverse carbon sources to fuel the tricarboxylic acid cycle (*15*–*17*). This adaptability enables tumor cells to withstand nutrient fluctuations and supports survival under therapeutic pressure, underscoring the importance of mitochondrial pathways in both tumor initiation and progression. Despite major advances in molecular classification and many therapeutic progresses in personalized medicine, effective targeted treatment options remain limited for most patients with non–small cell lung cancer (NSCLC) (*18*). The advent of allele-specific *KRAS* inhibitors has finally provided a long-awaited therapeutic strategy for *KRAS* mutant tumors and has more recently produced meaningful clinical responses (*19*, *20*). However, resistance to *KRAS* inhibition emerges rapidly in many patients (*21*), revealing an urgent need to identify complementary pathways that sustain tumor cell survival and limit therapeutic response.

Here, we studied the link between PINK1-dependent mitophagy, lung cancer development, and response to *KRAS*-targeted therapy. We first established that PINK1-dependent mitophagy is present in patients with NSCLC. Then, using preclinical models of lung cancers driven by the oncogenic *KRAS*^G12D^ and deficient Tp53, expressing the mitophagy reporter mito-QC, we demonstrated that mitophagy is increased in both preneoplastic lesions and lung adenocarcinoma (LUAD) and that PINK1-dependent mitophagy pathway is activated. Using genetic KO of PINK1, we established that mitophagy is essential for oncogene-driven transformation but not for the progression of already transformed tumors. Last, we revealed in vitro and in vivo that PINK1 depletion sensitized *KRAS*-driven lung cancer to targeted therapies.

## RESULTS

### Human NSCLC tumors have increased PINK1-dependent mitophagy

The role of autophagy in lung cancer development is well established, whereas the contribution of mitophagy remains less well defined. To investigate whether mitophagy is altered in human tumors, we first compared gene-expression profiles between nonlesional and tumor tissues from patients with LUAD, the most frequent form of NSCLC. This analysis revealed an enrichment of mitophagy-associated gene signatures among the most dysregulated pathways (Fig. 1, A and B). However, because mitophagy is largely controlled through posttranslational protein modifications, transcriptional changes alone are unlikely to accurately capture pathway activity. To more directly assess mitophagy activation, we examined the expression of ARIH1, an essential E3 ligase mediating mitophagy in lung cancer cells (*14*), and the phosphorylated form of RAB8A on Ser^111^ (P-Ser^111^ RAB8A), a biochemical readout of PINK1 kinase activity (*11*) as illustrated in fig. S1A, which shows that upon mitochondrial damage, PINK1 is stabilized and results in increase in P-Ser^111^ RAB8A and in P-Ser^65^–ubiquitin. The specificity of each antibody used is tested in lung cancer cell KO for PINK1. Across two independent LUAD cohorts analyzed by (phospho)proteomics, we observed significantly increased ARIH1 abundance and elevated P-Ser^111^ RAB8A levels in tumor tissues compared with nonlesional samples (Fig. 1C). Total RAB8A expression was either unchanged or decreased depending on the dataset analyzed (fig. S1B), indicating that the increase in P-Ser^111^ RAB8A is not due to elevated total protein levels but reflects PINK1 activation. However, PINK1 protein was not detected in these proteomic datasets and therefore could not be directly assessed in our analysis.

**Fig. 1.**
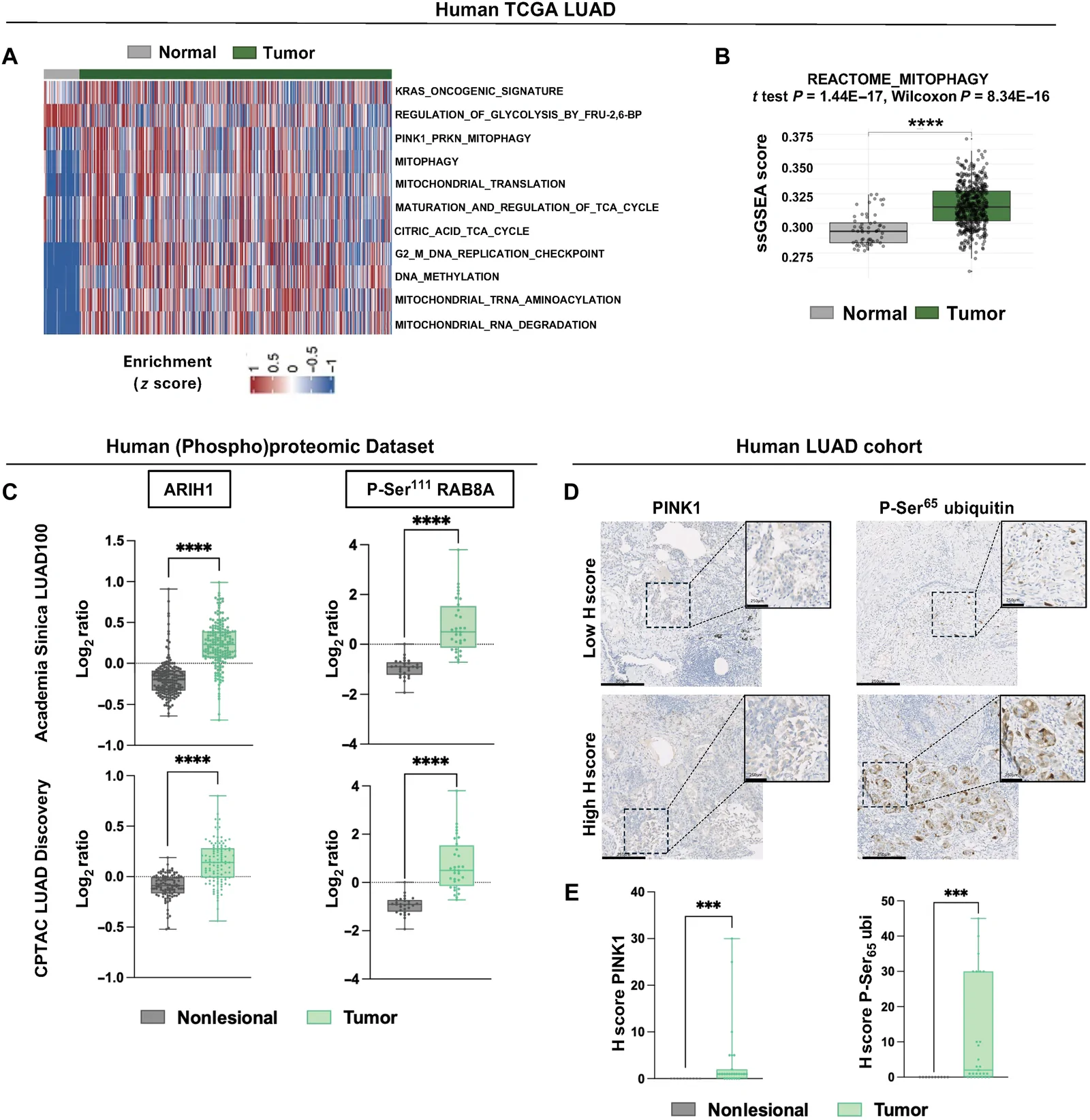
PINK1-dependent mitophagy occurs in patients with lung cancer. (**A**) Single-sample Gene Set Enrichment Analysis (ssGSEA) scores for selected Reactome pathways in TCGA LUAD samples. Heatmap represents the principal statistically significant pathways identified in tumor (*n* = 517) versus normal (*n* = 59) comparisons (FDR ≤ 0.05). ssGSEA scores were scaled by pathway (row-wise *z* score) to highlight relative differences across samples. (**B**) Reactome_Mitophagy ssGSEA profile between nonlesional and tumor samples from TCGA LUAD. (**C**) Analysis of ARIH1 and P-Ser^111^ RAB8A relative expression between normal adjacent and tumor tissues in published LUAD proteomic and phosphoproteomic databases from the NIH Proteomic Data Commons. Data are represented in Log2 ratio. Mann-Whitney unpaired *t* -test: ****P < 0.0001. (**D**) Illustration of IHC staining of PINK1 and P-Ser^65^ ubiquitin on formalin-fixed paraffin-embedded from patients bearing *KRAS*^G12C^ mutation. DAB-stained sections were imaged with a slide scanner microscope at 20× magnification. Scale bar, 250 μm. (**E**) Distribution of PINK1 and P-Ser^65^ ubiquitin expression scored by IHC staining on nonlesional and tumor areas (*n* = 10 and *n* = 27 respectively). Mann-Whitney unpaired *t* test: ***P = 0.0001 and ***P = 0.0002 (PINK1 and P-Ser^65^ ubiquitin respectively).

To further validate these findings and to enable assessment of PINK1 expression and activity in human tumor samples, we first validated the specificity of PINK1 detection tools in the human lung cancer cell line A549. Because endogenous PINK1 protein is rapidly degraded under basal conditions, transient mitochondrial depolarization with oligomycin/antimycin (O/A) was used solely as a technical control to stabilize PINK1 and validate the tools. As expected, O/A treatment increased PINK1 protein levels and kinase activity, as measured by phospho-ubiquitin (P-Ser^65^ ubiquitin) and phospho-RAB8A (P-Ser^111^ RAB8A). PINK1 knockout abolished detection of both PINK1 and P-Ser^65^ ubiquitin by immunoblots (fig. S1A), immunofluorescence (fig. S1, C and D), and immunohistochemistry (fig. S1, E and F), demonstrating the specificity of these assays toward the form of PINK1 regulating mitophagy. Using these validated reagents, we analyzed 10 adjacent noncancerous and 27 LUAD samples harboring *KRAS*^G12C^ mutations and quantified PINK1 expression and activity by IHC for P-Ser^65^ ubiquitin (Fig. 1, D and E). Tumor cells displayed markedly increased PINK1 expression and activity compared with adjacent noncancerous cells. Collectively, these data demonstrate that PINK1-dependent mitophagy is elevated in NSCLC.

### PINK1-dependent mitophagy is activated early and sustained throughout lung tumorigenesis

To examine the contribution of PINK1-dependent mitophagy to lung tumor development in vivo, we used a widely used tissue-specific and inducible *KRAS*^G12D^-driven genetically engineered mouse model (GEMM) of lung cancer, without (K model) or with Tp53 loss (KP model). As observed in human tissues, we first confirmed an enrichment of mitophagy-associated gene signatures among the most dysregulated pathways in these models when comparing tumor with nonlesional tissues (Fig. 2, A and B), supporting the suitability of these models to study the role of PINK1-dependent mitophagy in lung cancer initiation, progression, and therapeutic response.

**Fig. 2.**
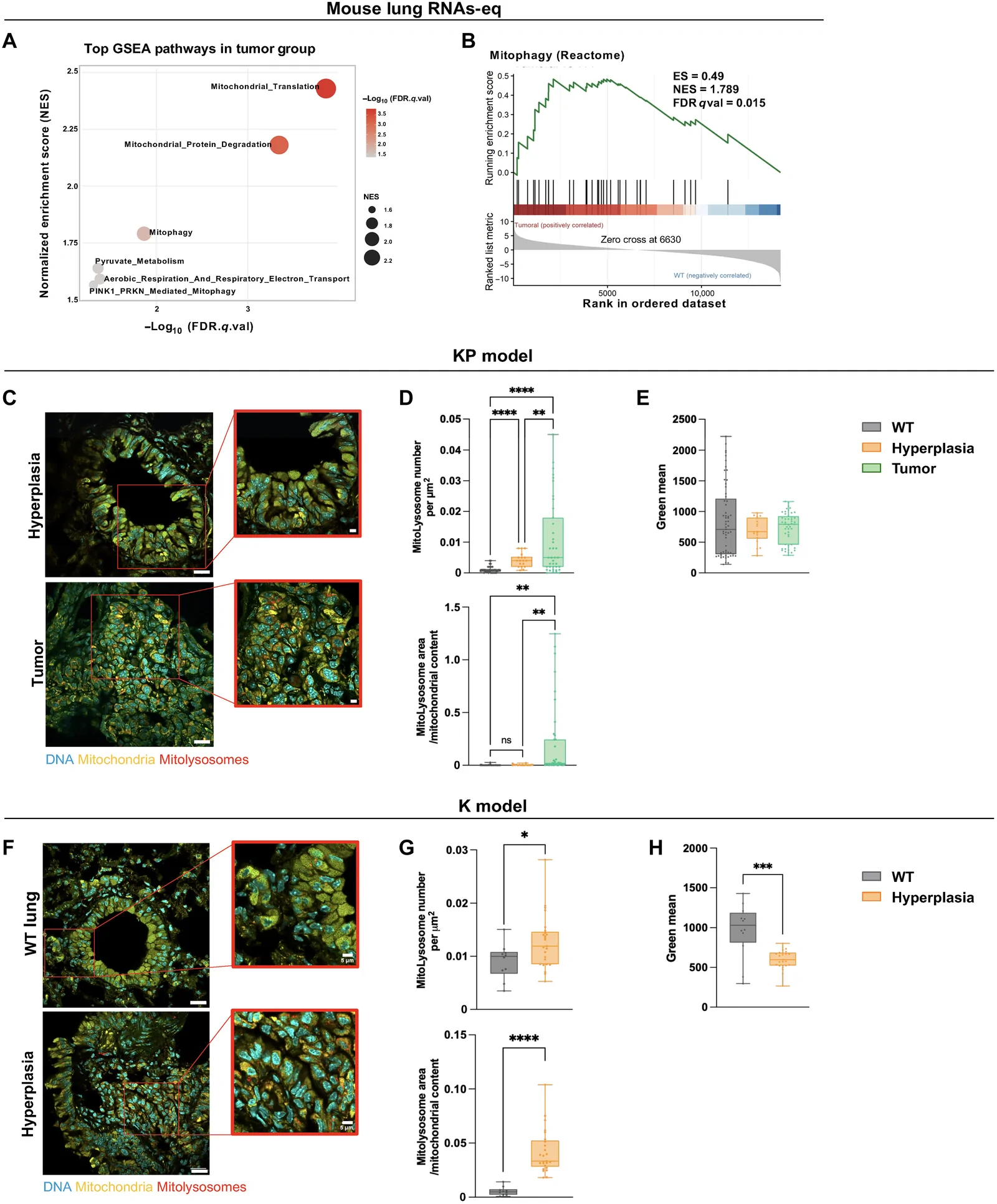
Mitophagy is activated during lung cancer progression in mice. (**A**) Top significant GSEA pathways in tumor compared to nonlesional samples after integrative RNA-seq analysis from published mouse lung cancer GEMM datasets. Datasets ID used for analysis are listed in Materials and Methods. (**B**) GSEA enrichment plot for REACTOME_Mitophagy signature in mouse lung cancer GEMM datasets. (**C**) mito-QC visualization (scale bars, 20 and 5 μm for higher magnification) and (**D**) Quantification of mitolysosomes number and size on frozen sections from lung KP-QC mice harvested at 12 to 15 weeks after tamoxifen injection [four WT-QC and six KP-QC mice per group, two to three zones per mouse, four to eight regions of interest (ROIs) per zone]. One-way analysis of variance (ANOVA) test: ****P < 0.0001, **P = 0.0079 (mitolysosome number hyperplasia versus tumor), **P = 0.0038 and 0.0044 (mitolysosome area/mitochondrial content WT versus tumor and hyperplasia versus tumor, respectively). (**E**) Corresponding quantification of mitochondrial mass. ns, not significant. (**F**) mito-QC visualization (scale bars, 20 and 5 μm for higher magnification) and (**G**) Quantification of mitolysosomes number and size on frozen sections from lung K-QC mice at 11 weeks post–tamoxifen injection (one WT and three K-QC mice, one to three zones per mouse, with two to five ROI per zone). Mann-Whitney unpaired *t* test: ****P < 0.0001, *P = 0.0496. Quantification was done with the mito-QC counter plugin in ImageJ software. (**H**) Corresponding quantification of mitochondrial mass. Mann-Whitney unpaired t-test: ***P = 0.0006.

To obtain a highly sensitive, cell-specific assessment of mitophagy, we crossed K and KP mice with mito-QC mice, which express a pH-sensitive fluorescent mitochondrial tandem reporter that allows in vivo evaluation of mitophagy (fig. S2, A and B) (*22*). *KRAS*^G12D^ induction (K-QC model) and Tp53 deletion (KP-QC model) was achieved by tamoxifen injection to activate the Cre recombinase expressed by club cell secretory protein (CCSP^+^) cells (fig. S2A). As expected, KP-QC mice developed hyperplasia and adenocarcinoma 12 to 15 weeks postinjection (fig. S2C). Using the mito-QC reporter, we observed an increase in both the number and size of mitolysosome in tumoral cells compared with healthy tissue (Fig. 2, C and D). Mitophagy was predominantly induced in E-cadherin–positive cells, a marker of lung normal and transformed epithelial cells in this model (fig. S2D). To exclude the possibility that the increased mito-QC signal simply reflected changes in mitochondrial abundance, we quantified the mean green fluorescent protein (GFP) fluorescence intensity of the mito-QC reporter, which provides a readout of total mitochondrial content independently of lysosomal delivery (*23*). No increase in mitochondrial content was detected in either hyperplastic lesions or adenocarcinomas compared with the surrounding nonlesional tissue (Fig. 2E), indicating that the increased mitophagy observed in tumor cells cannot be explained by an increase in mitochondrial mass. To further validate these findings using an independent approach, we quantified COX IV expression in the corresponding lesions. Consistent with the mito-QC analysis, COX IV levels remained unchanged or were slightly reduced during tumor progression (fig. S2, E and F), further supporting the conclusion that mitochondrial mass does not increase in our models. Similar results were obtained when mutant *KRAS*^G12D^ expression and Tp53 deletion was induced in lung cells via intratracheal delivery of Lenti-Cre particles (fig. S2, G and H).

We detected a small but significant increase in mitolysosome number even in hyperplastic lesions before full transformation (Fig. 2, C and D). To further validate this observation, we analyzed the K-QC model, which develops hyperplasia but rarely progresses to adenocarcinoma within this short post–tamoxifen window. This analysis confirmed a significant increase in both mitolysosome number and size in hyperplastic lesions independently of an increase in mitochondrial mass (Fig. 2, F to H), demonstrating that oncogene activation triggers mitophagy at the earliest stages of tumor initiation and that mitophagy intensifies as lesions advance toward malignancy, independently of the presence of Tp53.

To determine whether activation of the PINK1-dependent pathway correlates with the increase in mitophagy observed in vivo, we first analyzed publicly available single-cell RNA sequencing (scRNA-seq) datasets from K and KP lung cancer models collected at defined stages of tumor development—early (tumor initiation), intermediate, and late (established malignant tumors) following oncogene induction (*24*). Visualization of these datasets using t-SNE revealed that cells from early-stage lesions cluster separately from those in late-stage tumors, reflecting progressive transcriptional reprogramming during tumor evolution (fig. S3A). Mitophagy-associated gene signatures were further enriched in late-stage tumors (Fig. 3A and fig. S3A), consistent with progressive activation of this mitochondrial quality control during tumor progression.

**Fig. 3.**
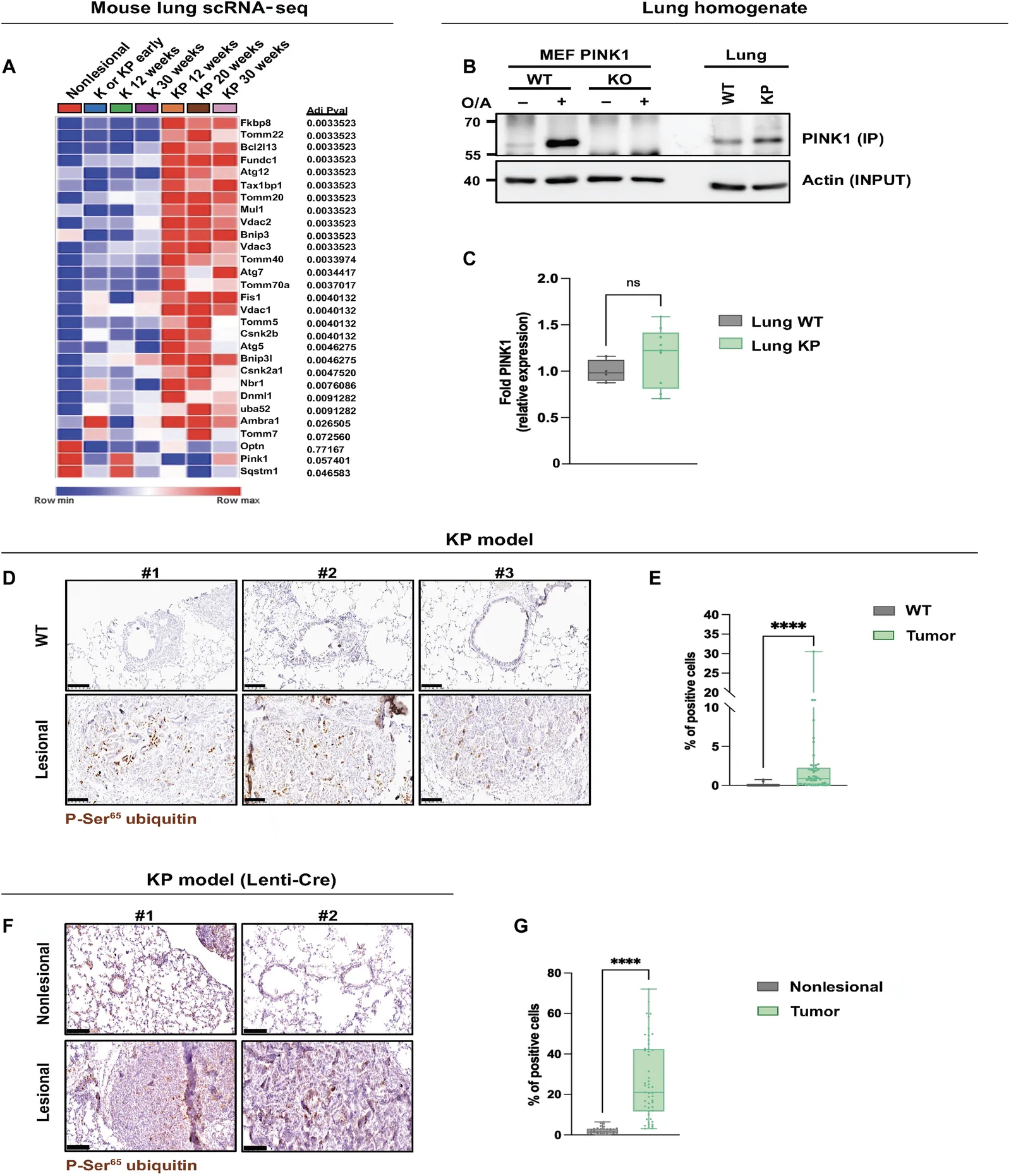
PINK1 activity is increased in tumors from KP lung mouse model. (**A**) Heatmap showing the mitophagy signature in tumoral cells from published scRNA-seq of lung GEMM carcinogenesis development over time (GSE152607). Data are expressed as *z* score with adjusted *P* value for each gene after comparison between group 1 (nonlesional, K or KP early and K 12 to 30 weeks) and group 2 (KP 12 to 20 to 30 weeks). (**B**) PINK1 immunoprecipitation on enriched lesions from KP-QC mice 12 weeks post–tamoxifen administration. (**C**) Semiquantification of PINK1 expression from immunoblots (*n* = 4 WT, *n* = 8 KP-QC). Unpaired *t* test with Welch correction; ns, not significant. (**D**) IHC staining of P-Ser^65^ ubiquitin on lung sections from KP-QC mice, 6 to 13 weeks post–tamoxifen administration. Photos were taken with a slide scanner microscope. Scale bar, 100 μm. (**E**) Quantification of P-Ser^65^ ubiquitin DAB staining using Qupath software (*n* = 4 WT mice, *n* = 9 KP-QC mice, 1 to 22 zones per mouse, 1 to 4 ROI per zone). Mann-Whitney unpaired *t* test, ****P < 0.0001. (**F**) P-Ser^65^ ubiquitin IHC-DAB staining on nonlesional and tumor lesions from KP-QC mice (Lenti-Cre model), 18 weeks post–intratracheal injection. Scale bar, 100 μm. (**G**) Corresponding quantification of P-Ser^65^ ubiquitin (*n* = 6 mice, 3 to 8 nonlesional and 4 to 12 tumor areas per mouse). Mann-Whitney unpaired *t* test, ****P < 0.0001.

To more deeply investigate PINK1-dependent mitophagy during lung cancer progression, we analyzed PINK1 expression and activity in tumors isolated from KP mice. Because assessing endogenous PINK1 protein in mice is technically challenging—owing to poor specificity of many commercially available antibodies—we rigorously validated the antibody used in this study (*25*) using PINK1-deficient mouse embryonic fibroblasts (MEFs), confirming its specificity (Fig. 3B and fig. S3B). Consistent with our observation, analysis of tumors tissues isolated from KP mice showed a trend toward increased PINK1 protein expression (Fig. 3, B and C, and fig. S3C). This was confirmed by IHC, where P-Ser^65^–ubiquitin levels were significantly increased in tumor areas from both tamoxifen (Fig. 3, D and E) and Lenti-Cre models (Fig. 3, F and G). IHC analyses further demonstrated that PINK1-dependent mitophagy occurs predominantly within cancer cells (fig. S3D). Together, these findings indicate that the K and KP GEMMs of lung cancers faithfully recapitulate key features of PINK1-driven mitophagy observed in human LUAD.

### PINK1 knockout limits cell transformation and reduces tumor burden upon oncogene induction

To dissect the role of PINK1-dependent mitophagy in tumor initiation and progression, we used the bicistronic pSECC vector, which enables simultaneous expression of Cre recombinase that will induce *KRAS*^G12D^ expression and deletion of Tp53, and CRISPR-Cas9–mediated targeting of PINK1 (fig. S4, A and B) (*26*). We first validated the efficiency of this construct to invalidate PINK1 in a Cre-reporter (GGC9) cell line (*26*). Upon infection with Cre-containing lentiviral particles, these cells become GFP^+^, thus enabling titration and sorting by flow cytometry. We confirmed robust Cre expression and efficient PINK1 knockout (fig. S4C). KP-QC mice were then intratracheally instilled with the Lenti-Cre pSECC vectors expressing either a control single guide RNA (sgRNA) or an sgRNA-targeting PINK1. Seventeen weeks after injection, mice were euthanized and lungs were analyzed. Strikingly, PINK1 depletion led to a profound reduction in tumor burden as evidenced by a marked decrease in the number and overall size of lung lesions (Fig. 4, A and B). Analysis of the mito-QC reporter in these mice further revealed a significant decrease in mitolysosome formation (Fig. 4, C and D), indicating that loss of PINK1 impairs mitophagy in vivo. Together, these results demonstrate that PINK1 depletion not only suppresses mitophagy but also restricts *KRAS*-driven lung tumor development in vivo.

**Fig. 4.**
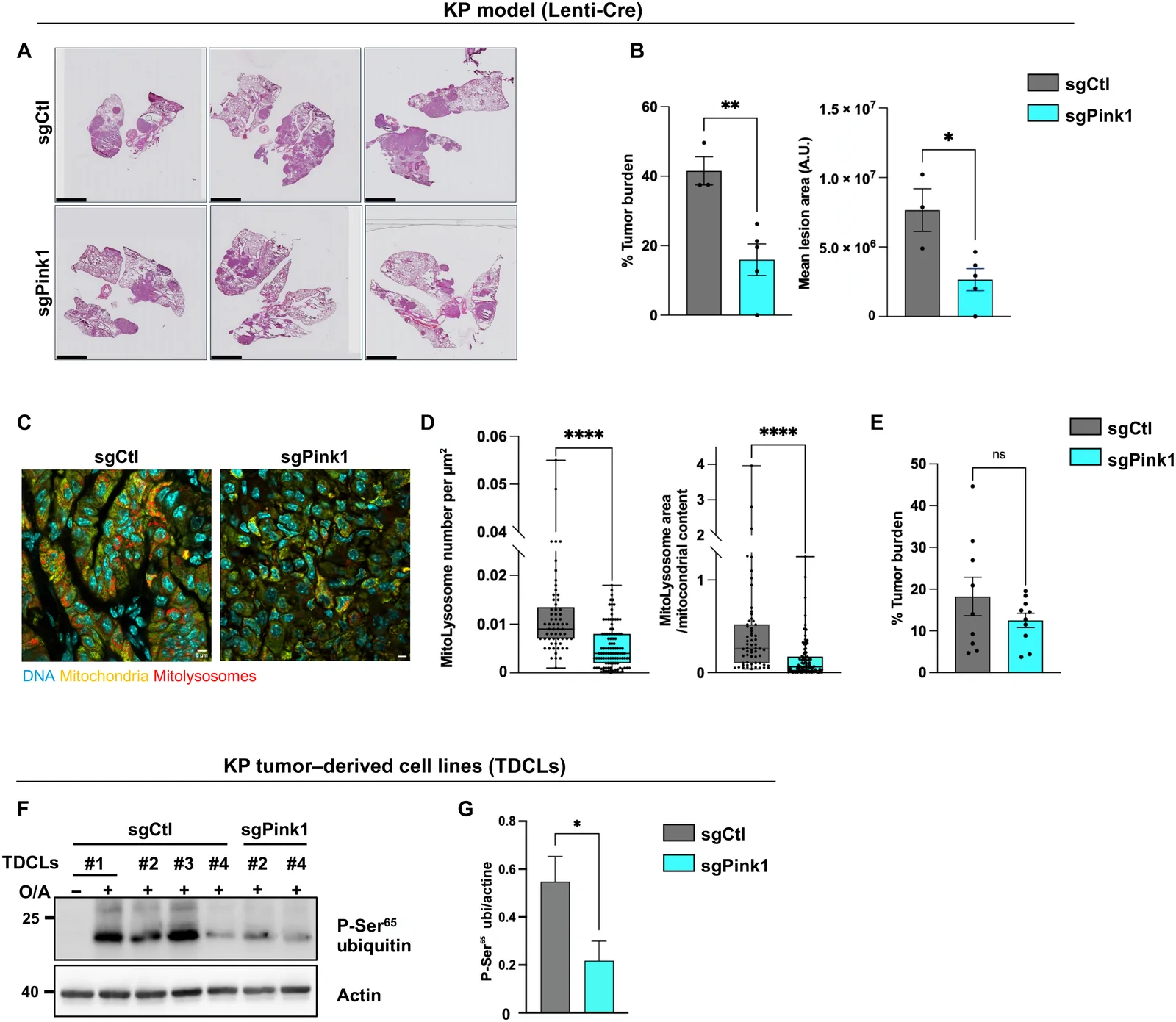
Pink1 deletion inhibits lung tumor progression in mice. (**A**) Hematoxylin and eosin staining on frozen lung sections from KP-QC mice, 17 weeks after intratracheal administration of either pSECC-sgCtl or pSECC-sgPink1, and corresponding (**B**) tumor burden (%) and mean lesions’ area evaluation. Photos were taken with a slide scanner microscope at 20× magnification. Scale bar, 2.5 mm. Data are represented as means ± SEM (*n* = 3 sgCtl mice, *n* = 5 sgPink1 mice). Unpaired *t* test. *P = 0.0303, **P = 0.0091. (**C**) mito-QC visualization on representative frozen lung sections and corresponding (**D**) quantification of mitolysosomes (number and size) of KP-QC mice, 17 weeks after intratracheal administration (*n* = 3 sgCtl mice, *n* = 5 sgPink1 mice, four to five tumoral zones per mice, five to six ROIs per zone). Mann-Whitney unpaired *t* test, ****P < 0.0001. Scale bar, 20 μm. (**E**) Tumor burden (%) from KP-QC mice, 12 weeks after intratracheal administration (*n* = 9 sgCtl mice, *n* = 10 sgPink1 mice). Unpaired *t* test; ns, not significant. (**F**) Western blot of TDCLs KP-sgCtl and KP-sgPink1, treated in vitro with either vehicle or 1 μM oligomycin and 1 μM antimycin A for 24 hours. (**G**) Corresponding semiquantification of P-Ser^65^ ubiquitin signal from OA-treated condition, normalized to actin, using ImageJ software. Data are represented as means ± SEM, *n* = 11 KP-sgCtl tumors and *n* = 3 KP-sgPink1 tumors. Unpaired *t* test with Welch’s correction, *P = 0.035. A.U., arbitrary units.

Because mitophagy induction was already evident in early hyperplastic lesions (Fig. 2, F and G), we repeated the experiment and analyzed mice 12 weeks after Lenti-Cre instillation, a stage at which most lesions remain at an early preneoplastic stage. In this earlier time window, PINK1 loss also tended to reduce lesion burden (Fig. 4E). To further assess this effect, we generated tumor-derived cell lines (TDCLs) from KP-sgCtl and KP-sgPink1 tumors 10 to 11 weeks after oncogene induction. Whereas 16 of 17 KP-sgCtl lesions yielded robustly propagating TDCLs, only three TDCLs could be successfully established from 5 KP sgPink1 nodules, indicating a markedly reduced capacity of PINK1-deficient lesions to give rise to sustained in vitro cultures (fig. S4D). These data also confirmed that the number of macroscopic lesions obtained in KP-sgPink1 lung, 10 weeks after injection is strongly reduced compared to sgCtl KP lung. Analysis of PINK1 activity in these TDCLs confirmed efficient knockout, as KP-sgPink1 cell lines showed a pronounced reduction in P-Ser^65^ ubiquitin levels (Fig. 4, F and G, and fig. S4E). Together, these findings suggest that PINK1-dependent mitophagy is particularly important during the earliest steps of oncogene-driven transformation.

To directly test this hypothesis, we isolated primary MEFs (pMEFs) from KP-QC mice. As expected, MEFs transduced with a control lentiviral vector (Lenti-GFP) did not proliferate under two-dimensional (2D) culture conditions. In contrast, MEFs transduced with lentiviral pSECC-sgCtl vector efficiently expressed *KRAS*^G12D^ and lost Tp53 (fig. S5, A and B), resulting in robust colony formation (Fig. 5, A and B). Transformed cells display increased PINK1 activity compared to pMEFs, and PINK1 knockout markedly reduced P-Ser^65^ ubiquitin levels (fig. S5, C and D) and caused a significant reduction in colony number, demonstrating that PINK1-dependent mitophagy is required for oncogenic *KRAS*/Tp53-driven cellular transformation.

**Fig. 5.**
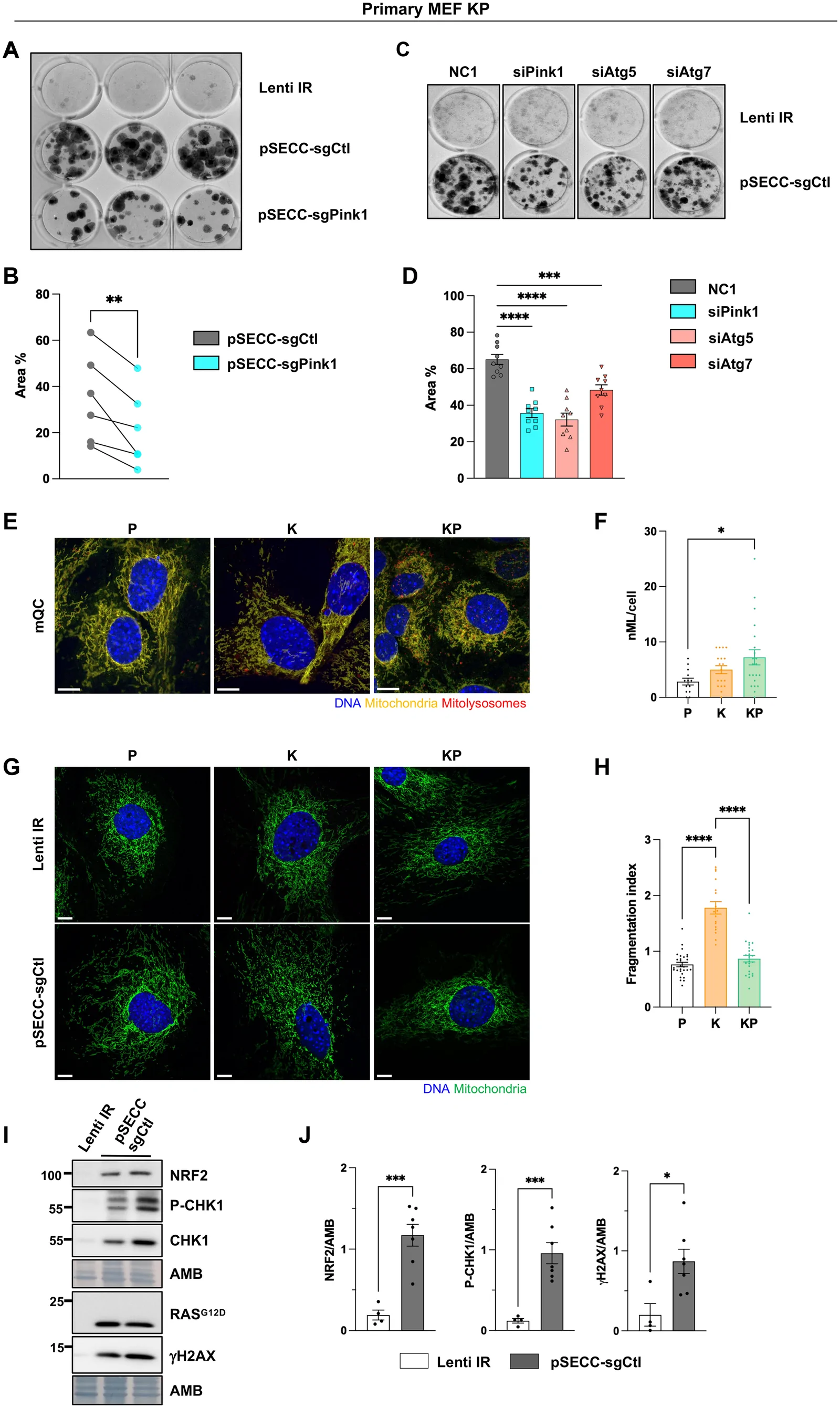
Oncogenic *KRAS*^G12D^ and p53 deletion promote PINK1-dependent mitophagy that supports cellular transformation. (**A**) 2D clonogenic assay with KP-QC pMEFs transduced with lentiviral particles containing pSECC vectors. (**B**) Corresponding quantification of colony area using the colony area plugin in ImageJ software. Data are represented as area percentage, six independent experiments. Paired *t* test **P = 0.0096. (**C**) 2D clonogenic assay with KP-QC pMEFs, transfected with siRNA targeting Pink1, Atg5, and Atg7 and transduced with lentiviral particles containing pSECC vector. (**D**) Corresponding quantification of colony area. Data are represented as means ± SEM, two independent experiments. One-way ANOVA, ****adj. *P*val <0.0001, ***adj. *P*val = 0.0009 (NC1 versus siPink1, siAtg5, and siAtg7, respectively). NC1: siRNA negative control. (**E**) mito-QC visualization (40× magnification and scale bar, 10 μm) and corresponding quantification (**F**) in P-QC, K-QC, and KP-QC pMEFs 10 days after transduction with lentiviral particles containing pSECC vector. Data are represented as means ± SEM, *n* = 13, 18, and 23 cells analyzed for P-QC, K-QC, and KP-QC, respectively. One-way ANOVA, * adj. *P* val = 0.0375. (**G**) Confocal microscopy of mitochondrial network of P-QC (P), K-QC (K), and KP-QC (KP) pMEFs 8 to 10 days after transduction with either irrelevant or pSECC-sgCtl lentiviral particles. 40× magnification; scale bar, 10 μm. (**H**) Corresponding quantification of fragmentation index. Data are represented as means ± SEM, *n* = 29, 17, 23 cells analyzed for P-QC, K-QC, and KP-QC, respectively. Brown-Forsythe and Welch ANOVA test, **** adj. *P* val < 0.0001. (**I**) NRF2, P-CHK1, CHK1, RASG12D, and γH2AX protein expression on total lysate from KP-QC pMEF 10 days after transduction with either irrelevant or pSECC-sgCtl lentiviral particles. (**J**) Corresponding quantification from the immunoblots in (I). Data are represented as means ± SEM, *n* = 4 Lenti IR and *n* = 7 pSECC-sgCtl biological replicates, unpaired *t* test with Welch correction, ***P = 0.0001, *P = 0.0113, ***P = 0.0005 (NRF2, γH2AX, and P-CHK1 respectively).

To further support our results, we investigated whether inhibition of the core autophagy machinery phenocopies PINK1 depletion during oncogenic transformation. To this end, pMEFs were transduced with the KP-inducing pSECC vector and simultaneously transfected with control, Pink1, Atg5, or Atg7 small interfering RNAs (siRNAs). Similar to PINK1 depletion, silencing either Atg5 or Atg7 significantly reduced colony formation following *KRAS*^G12D^ activation and Tp53 deletion (Fig. 5, C and D, and fig. S5E), demonstrating that an intact autophagy pathway is required for efficient oncogenic transformation. These findings indicate that the protumorigenic function of PINK1 during cellular transformation is largely mediated through its role in mitophagy/autophagy rather than through mitophagy-independent functions.

To determine whether this mitophagy response specifically depends on the combined oncogenic alterations, we quantified mitophagy using the mito-QC reporter in MEFs carrying *KRAS*^G12D^ activation alone (K), Tp53 deletion alone (P), or the combined KP genotype. Whereas either genetic alteration alone induced only a modest increase in mitophagy, the combined KP genotype resulted in a markedly stronger response (Fig. 5, E and F), indicating that robust mitophagy is preferentially associated with the combined activation of oncogenic KRAS and loss of Tp53.

We next investigated the mitochondrial phenotype associated with transformation. Mitochondrial fragmentation was most pronounced following *KRAS*^G12D^ activation alone, whereas the combined KP genotype did not further increase mitochondrial fragmentation (Fig. 5, G and H), consistent with the absence of increased dynamin-related protein 1 (DRP1) Ser^616^ phosphorylation (equivalent to murine phospho-Ser^592^, fig. S5F). Although mitochondrial fission can facilitate mitophagy, these processes are not obligatorily coupled, and DRP1-dependent fission is not strictly required for mitophagy in all cellular contexts (*27*). Thus, the enhanced mitophagy observed in KP cells cannot simply be explained by increased mitochondrial fragmentation.

Last, we investigated whether the combined activation of oncogenic *KRAS* and Tp53 loss generates cellular stress signals associated with mitophagy induction. KP transformation resulted in increased NRF2 expression together with enhanced CHK1 phosphorylation and γH2AX accumulation, indicating reactive oxygen species (ROS)–dependent activation of the DNA damage response (Fig. 5, I and J). Treatment with the ROS scavenger *N*-acetylcysteine (NAC) markedly reduced NRF2 induction, CHK1 phosphorylation, and γH2AX accumulation (fig. S5G), demonstrating that oxidative stress acts upstream of this response. Together, these findings indicate that combined oncogenic *KRAS* activation and Tp53 loss induce ROS-dependent activation of the DNA damage response, providing a mechanistic framework consistent with the enhanced mitophagy observed during oncogenic transformation.

### PINK1-dependent mitophagy is dispensable for proliferation and tumor growth once cells are fully transformed

To determine whether PINK1-dependent mitophagy remains necessary after malignant transformation, we generated KP-sgCtl and KP-sgPink1 from a KP cell line established from tamoxifen-induced lesions. As expected, O/A treatment robustly induced PINK1-dependent mitophagy in control KP cells, whereas PINK1-deficient cells showed markedly reduced induction of P-Ser^65^ ubiquitin or P-Ser^111^ RAB8A (Fig. 6, A and B) and mitophagy (Fig. 6C). We next assessed cell proliferation and observed only a modest reduction in growth rate in PINK1-deficient cultures over time (Fig. 6D). To evaluate whether this subtle in vitro phenotype translated into a functional impact in vivo, we injected these cells subcutaneously into syngeneic immunocompetent mice. Tumor growth kinetics (Fig. 6E) and final tumor weights at end point (Fig. 6F) were indistinguishable between control and PINK1-deficient groups. We confirmed that PINK1-deficient tumors maintained the expected loss of PINK1 activity, as indicated by the absence of P-Ser^65^ ubiquitin protein expression (Fig. 6, G and H).

**Fig. 6.**
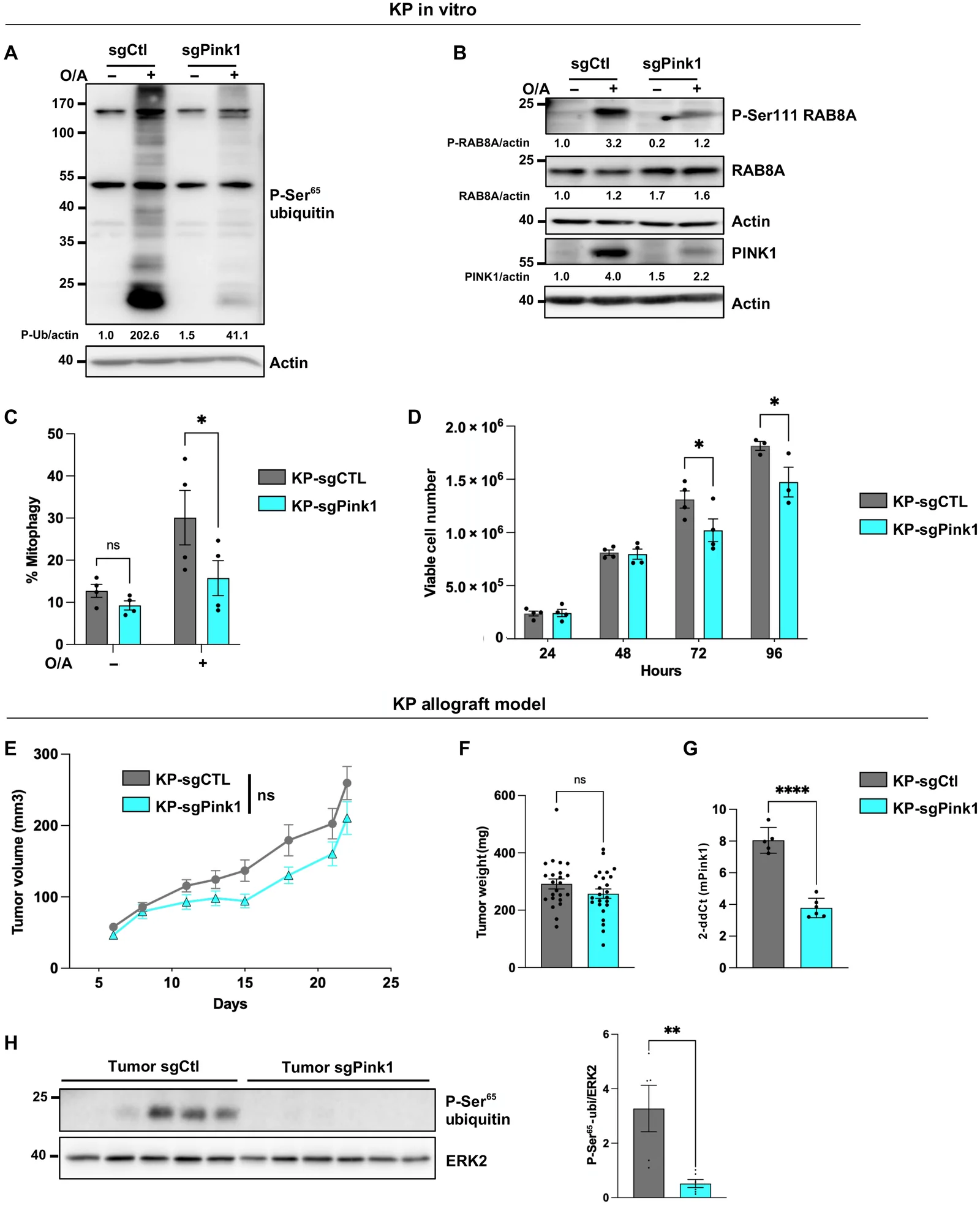
PINK1-dependent mitophagy occurs in lung cancer cells in vitro without impacting tumor growth. (**A** and **B**) P-Ser^65^ ubiquitin, P-Ser^111^ RAB8A and total RAB8A expression on total lysate from KP cell line either Ctl (KP-sgCtl) or invalidated for PINK1 (KP-sgPink1) treated with oligomycin and antimycin A (5 and 4 μM, respectively) for 24 hours. (**C**) Mito-mkeima assay on KP-sgCtl and KP-sgPink1 cell lines treated 48 hours with either vehicle or oligomycin and antimycin A (1 μM each). Data are represented as means ± SEM, *n* = 2 independent experiments, two technical replicates per assay. Two-way ANOVA, *adj. *P* val = 0.0485. (**D**) KP-sgCtl and KP-sgPink1 cell growth kinetic. Data are represented as means DAPI-negative cells ± SEM, *n* = 4 independent experiments. Two-way ANOVA, *adj. *P* val = 0.0239 and 0.0209 (72 and 96 hours, respectively). (**E**) Tumor growth monitoring in syngeneic mice bearing KP-sgCtl and KP-sgPink1 subcutaneous tumors. Data are represented as means ± SEM, *n* = 23 KP-sgCtl and *n* = 24 KP-sgPink1. Two-way ANOVA; ns, not significant. (**F**) Tumor weight at end point of allograft tumors from (E). Data are represented as means ± SEM. Unpaired *t* test; ns, not significant. (**G**) Pink1 mRNA expression from ex vivo RT-qPCR analysis of KP-sgCtl (*n* = 5) and KP-sgPink1 (*n* = 6) tumors. Data are represented as means ± SEM. Unpaired *t* test, ****P < 0.0001. (**H**) Evaluation and quantification of P-Ser^65^ ubiquitin expression by Western blot from KP-sgCtl (*n* = 5) and KP-sgPink1 (*n* = 6) tumor protein extracts.

To directly assess whether the requirement for PINK1 is stage dependent, we compared the consequences of PINK1 depletion during oncogenic transformation and after stable transformation had been established. Whereas PINK1 depletion significantly impaired colony formation during *KRAS*^G12D^ /Tp53-driven transformation (Fig. 5, A to D), it no longer affected the clonogenic capacity of established KP-transformed MEFs maintained in long-term culture (fig. S5H). Together, these findings indicate that PINK1-dependent mitochondrial quality control is specifically required during the early adaptation to oncogenic transformation but becomes largely dispensable once transformed cells are established.

### PINK1-dependent mitophagy supports tumor cell survival under *KRAS* inhibition

Given the recent clinical deployment of *KRAS*-targeted inhibitors for *KRAS*^G12C^-mutant NSCLC [sotorasib and adagrasib, (*28*)] and the rapid emergence of therapeutic resistance (*29*, *30*), we asked whether mitochondrial quality control, specifically PINK1-dependent mitophagy, modulates the response to *KRAS* inhibition. MRTX1133 has recently been shown to promote mitophagy in pancreatic ductal adenocarcinoma cell line (*31*). We first treated KP-sgCtl and KP-sgPink1 cells with sotorasib. Consistent with their *KRAS*^G12D^ genotype, neither cell line exhibited any detectable response to this *KRAS*^G12C^-specific inhibitor, confirming that loss of PINK1 does not nonspecifically sensitize cells to unrelated targeted therapies (fig. S6A). In contrast, treatment with MRTX1133, a *KRAS*^G12D^ inhibitor used in PDAC preclinical models (*32*, *33*) and recently in phase 1/2 for solid tumors (NCT05737706), produced a dose-dependent inhibition of cell growth, which was significantly enhanced following PINK1 depletion. This phenotype was confirmed using both flow cytometry–based proliferation assays (fig. S6B) and long-term clonogenic assays (Fig. 7, A and B). MRTX1133 suppressed extracellular signal–regulated kinase (ERK) phosphorylation to a similar extent in KP-sgCtl and KP-sgPink1 cells (fig. S6, C and D), indicating that the increased sensitivity of PINK1-deficient cells is independent of *KRAS* target inhibition.

**Fig. 7.**
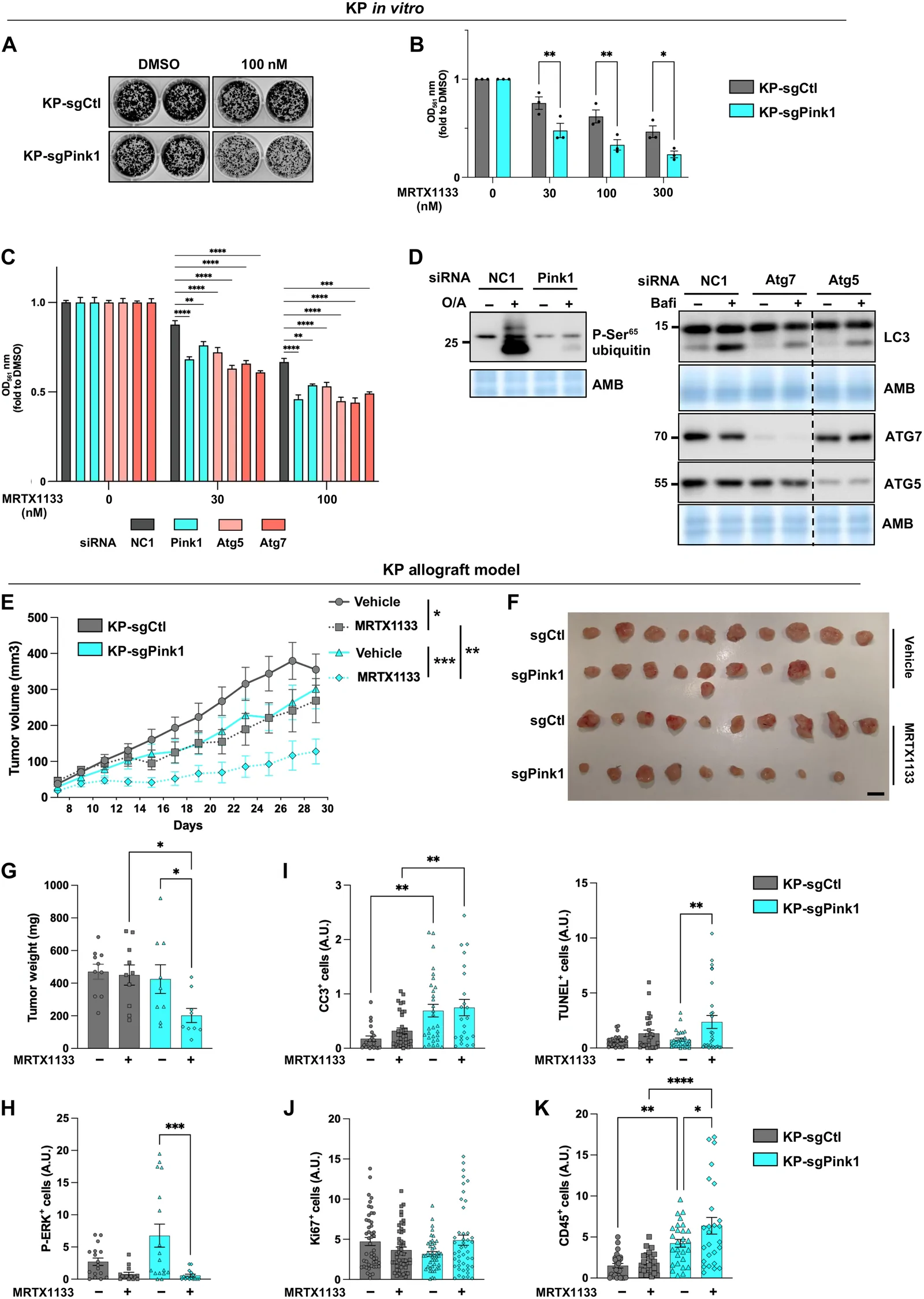
Pink1 knockout sensitizes KP cells and tumors to *KRAS*^G12D^ inhibition. (**A**) Representative 2D clonogenic assay of KP-sgCtl and KP-sgPink1 cell lines treated with MRTX1133 for 5 days. (**B**) Corresponding quantification of 2D clonogenic assays, expressed as fold change relative to dimethyl sulfoxide (means ± SEM), *n* = 3 independent experiments. 2-way ANOVA, **adj. *P* val = 0.0058, **adj. *P* val = 0.0044, *adj. *P* val = 0.0235 (for 30, 100, and 300 nM, respectively). (**C**) Quantification of clonogenic assays performed on KP cells transfected with siRNAs targeting Pink1, Atg5, or Atg7, followed by 5-day treatment with MRTX1133, *n* = 3 independent experiments. (**D**) Validation of Pink1, Atg5, and Atg7 knockdown in KP cell line. For Pink1, cells were treated 24 hours with oligomycin and antimycin A (1 μM each). For Atg5 and Atg7, cells were treated 2 hours with bafilomycin A (100 nM). Nonadjacent lanes from the same blot are separated by dashed lines. (**E**) Growth curves of KP-sgCtl and KP-sgPink1 subcutaneous tumors treated with vehicle or MRTX1133 (3 mg/kg). Data are represented as means ± SEM, KP-sgCtl vehicle (*n* = 10), KP-sgCtl MRTX1133 (*n* = 10), KP-sgPink1 vehicle (*n* = 9), KP-sgPink1 MRTX1133 (*n* = 9). Two-way ANOVA, *adj. *P* val = 0.048, **adj. *P* val = 0.0017, ***adj. *P* val = 0.0002. (**F**) Images of excised tumors; scale bar, 1 cm, and (**G**) corresponding tumor weight. Data are represented as means ± SEM. One-way ANOVA with Sidak for multiple testing conditions, *adj. *P* val = 0.035 and 0.015 (KP-sgPink1 vehicle versus MRTX1133 and KP-sgCtl MRTX1133 versus KP-sgPink1 MRX1133, respectively). (**H** to **K**) Quantification of phospho-ERK (H), cleaved caspase 3 (CC3) and TUNEL (I), Ki67 (J), and CD45 (K) normalized to tumor weight. Three to 10 mice per group and five ROIs per mouse were analyzed. Data are represented as arbitrary units (A.U.) ± SEM, one-way ANOVA, *P < 0.05, **P < 0.01, and ***P < 0.001.

We next investigated whether this phenotype reflected a broader requirement for the autophagy/mitophagy machinery. Similar to PINK1 depletion, transient silencing of the core autophagy regulators ATG5 or ATG7 significantly potentiated the antiproliferative effects of MRTX1133 (Fig. 7, C and D), indicating that an intact autophagy pathway contributes to the adaptive response to *KRAS* inhibition. To further assess the generality of this observation, we extended our analysis to LLC1 lung cancer cells, which harbor a distinct oncogenic *KRAS* allele and are therefore sensitive to the *KRAS*^G12C^ inhibitor sotorasib. Consistent with our observations in KP cells, PINK1 depletion significantly enhanced the response of LLC1 cells to *KRAS* inhibition (fig. S6, E and F), demonstrating that this vulnerability is conserved across distinct *KRAS*-driven lung cancer models, different *KRAS* oncogenic alleles, and independent *KRAS*-targeted therapies.

We next validated these findings in vivo. KP-sgCtl and KP-sgPink1 cells were implanted subcutaneously into syngeneic immunocompetent mice, which subsequently received daily MRTX1133 treatment (fig. S6G). While control tumors exhibited only a modest response to *KRAS* inhibition, PINK1-deficient tumors displayed a marked reduction in growth (Fig. 7, E to G). Immunohistochemical analysis of tumor sections confirmed efficient inhibition of *KRAS* signaling, as evidenced by equivalent suppression of phospho-ERK in both genotypes following MRTX1133 treatment (Fig. 7H). Cleaved caspase-3 staining revealed increased apoptosis in PINK1-deficient tumors both at baseline and following treatment, whereas terminal deoxynucleotidyl transferase–mediated deoxyuridine triphosphate nick end labeling (TUNEL) staining demonstrated a significant increase in cell death specifically in MRTX1133-treated PINK1-deficient tumors (Fig. 7I). In contrast, no significant differences were detected in Ki67 staining (Fig. 7J). This likely reflects the marked increase in CD45^+^ immune cell infiltration observed in PINK1-deficient tumors, which was further enhanced by MRTX1133 treatment (Fig. 7K). Because Ki67 was quantified across whole tumor sections, proliferating immune cells likely contributed to the overall Ki67 signal and masked a reduction in tumor cell proliferation despite the increased tumor cell death.

Collectively, these findings identify PINK1-dependent mitophagy as a critical adaptive pathway that promotes tumor cell survival during *KRAS*-targeted therapy. Pharmacological inhibition of *KRAS* remains equally effective at suppressing downstream signaling in the presence or absence of PINK1; however, loss of PINK1-dependent mitochondrial quality control markedly increases tumor cell susceptibility to *KRAS* inhibition, establishing mitophagy as a promising therapeutic vulnerability in *KRAS*-driven lung cancer.

## DISCUSSION

Although mitophagy is increasingly recognized as a critical regulator of cellular fitness, its precise contribution to tumor initiation and response to therapy remains unclear. By combining analysis of human lung tumors with direct in vivo visualization of mitophagy using mito-QC reporter mice in *KRAS*^G12D^ activation (K) and Tp53 deletion (KP) models, our study provides the demonstration that physiopathological mitophagy is engaged during the earliest stages of primary lung tumor development in the absence of exogenous mitochondrial stress. We further show that PINK1-dependent mitophagy promotes oncogenic transformation but becomes largely dispensable once tumor cells are established. Last, we identify PINK1-dependent mitochondrial quality control as a determinant of the response to *KRAS*-targeted therapy. Together, these findings establish PINK1-dependent mitophagy as a stage-specific regulator of *KRAS*-driven lung tumorigenesis and identify mitochondrial quality control as a therapeutically actionable vulnerability.

While global autophagy inhibition has been shown to impair tumor growth by promoting the accumulation of damaged mitochondria, such models do not distinguish mitophagy from other forms of selective autophagy (*34*). Prior work has implicated selective mitophagy pathways in tumor initiation: For example, *KRAS*-dependent up-regulation of NIX (BNIP3L) promotes mitophagy in a pancreatic cancer model, and NIX deletion delays tumor progression (*35*). In addition, loss of PINK1 or PARK2 in *KRAS*-driven pancreatic GEMMs accelerates tumorigenesis through dysregulation of mitochondrial iron metabolism and immunometabolic signaling (*36*) illustrating the context-dependent functions of mitochondrial quality control in cancer. Our study extends these observations by providing direct in vivo evidence that PINK1-dependent mitophagy is activated from the earliest preneoplastic stages of lung tumorigenesis and functionally contributes to oncogenic transformation. Genetic inhibition of the core autophagy machinery through Atg5 or Atg7 knockdown closely phenocopied PINK1 loss during both cellular transformation and the response to *KRAS* inhibition. Although we cannot formally exclude additional mitophagy-independent functions of PINK1, these findings strongly support the conclusion that the biological effects described here are primarily linked to its role in mitochondrial quality control.

Several clinical and transcriptomic studies have reported that PINK1 expression correlates with poor prognosis in NSCLC (*37*–*39*). However, interpretation of these studies has remained challenging because mitophagy is predominantly regulated through posttranslational mechanisms, and PINK1 activity cannot be inferred from transcript abundance alone. By integrating transcriptomic analyses with phosphoproteomic datasets, biochemical measurements of PINK1 kinase activity, and in vivo mito-QC imaging, our study provides a functional assessment of mitophagy rather than relying solely on gene-expression signatures. These complementary approaches demonstrate that PINK1 kinase activity and mitophagy are both increased during human lung tumorigenesis, thereby establishing a direct link between activation of the PINK1 pathway and disease initiation.

The key role played by PINK1 during transformation may reflect the profound mitochondrial stress associated with the acquisition of the transformed phenotype as it was shown that DRP1, a central component of the fission-fusion machinery, is required for RAS-induced transformation (*40*). We demonstrated that combined *KRAS* activation and Tp53 loss induce ROS-dependent activation of the DNA damage response, providing a plausible cellular context for the induction of mitophagy. These observations are consistent with recent evidence linking DNA damage response signaling to mitophagy activation (*41*) and support a model in which mitochondrial quality control constitutes an adaptive response to oncogenic stress.

Mitophagy may therefore act as a buffering mechanism that allows cells to tolerate mitochondrial stress, sustain mitogen-activated protein kinase (MAPK) signaling, and undergo oncogene-driven transformation. Consistent with this model, we established that loss of PINK1 resulted in a reduction in transformation efficiency in vitro and in vivo. In contrast, once cells were fully transformed, PINK1 became largely dispensable as PINK1-deficient tumor cells expanded in vitro and formed subcutaneous tumors with kinetics similar to controls. This finding parallels observations in human A549 xenografts, where PINK1 knockdown did not impair tumor growth in immunodeficient mice (*42*). Together, these findings indicate that PINK1-dependent mitochondrial quality control is particularly important during the initial adaptation to oncogenic stress, whereas transformed cells progressively acquire compensatory mechanisms that allow maintenance of mitochondrial homeostasis independently of PINK1. One possibility is that established tumors increasingly rely on alternative mitochondrial quality-control pathways, including receptor-mediated mitophagy, although this remains to be directly investigated. This stage-dependent model also reconciles our functional observations with the persistent mitophagy signatures detected in human LUAD. Elevated mitophagy in established tumors does not necessarily imply continued dependency on PINK1. Rather, it likely reflects sustained activation of mitochondrial quality-control programs that may be maintained through both PINK1-dependent and PINK1-independent mitophagy pathways according to the stage of disease and tumor context.

Recent work further supports the concept that mitochondrial and metabolic stress responses shape the therapeutic vulnerabilities of *KRAS*-mutant tumors. In particular, *KRAS* inhibition induces pronounced mitochondrial dysfunction and oxidative stress, triggering a compensatory program centered on macroautophagy and glutathione (GSH) biosynthesis (*43*). These pathways cooperate to maintain redox balance and mitochondrial integrity during *KRAS* blockade, allowing tumor cells to withstand the metabolic burden imposed by loss of oncogenic signaling. Disrupting either autophagic flux or GSH synthesis markedly sensitized *KRAS*-mutant cells to *KRAS* inhibitors in vitro and in vivo. Our findings extend this model by identifying PINK1-dependent mitophagy as an additional and mechanistically distinct component of this adaptive mitochondrial stress response. Genetic inhibition of the core autophagy machinery through ATG5 or ATG7 depletion closely reproduced the effects of PINK1 loss, supporting the conclusion that the enhanced sensitivity to *KRAS* inhibition primarily reflects disruption of mitochondrial quality control rather than other putative functions of PINK1.

*KRAS* mutations represent one of the most prevalent oncogenic lesions in LUAD and underpin a transcriptional and metabolic program that sustains uncontrolled growth, promotes immune evasion, and drives metastatic progression (*44*). Despite the recent development of allele-specific *KRAS* inhibitors, therapeutic responses in patients remain incomplete and transient. As highlighted in recent work, *KRAS*-mutant tumors rapidly engage diverse adaptive mechanisms to bypass pathway inhibition, including reactivation of upstream receptor tyrosine kinases, restoration of MAPK signaling through alternative RAS-pathway nodes, and rewiring of PI3K-AKT survival pathways (*44*). In parallel, tumor cells undergo notable state transitions—shifts in differentiation, chromatin remodeling, and metabolic reprogramming—that collectively blunt the impact of *KRAS* blockade. These multifaceted escape routes underscore the intrinsic plasticity of *KRAS*-driven cancers and explain why resistance emerges almost universally, even in the presence of potent inhibitors. Our findings introduce mitochondrial quality control as an additional and previously underappreciated layer of this adaptive landscape.

PINK1-deficient tumors also displayed increased CD45-positive immune cell infiltration, which was further enhanced following *KRAS* inhibition. Although the contribution of the immune microenvironment was not directly investigated in the present study, these observations suggest that altered mitochondrial quality control may influence tumor-immune interactions during therapeutic response. Future studies are ongoing to determine whether modulation of mitophagy directly contributes to antitumor immunity in *KRAS*-driven lung cancer.

In conclusion, our findings reveal that PINK1-dependent mitophagy is a key determinant of early *KRAS*-driven lung tumor development and a mediator of adaptive resistance to *KRAS* inhibition. These results suggest that targeting mitochondrial quality control—alone or in combination with *KRAS*-directed therapies—may offer a promising strategy to restrict malignant transformation and enhance treatment efficacy in *KRAS*-mutant lung cancer.

## MATERIALS AND METHODS

### Cell culture conditions and treatment

*KRAS*^G12D/^+^^; p53^−/−^ (KP) TDCL was established by cutting up lung tumors from CKP-QC mice (8 weeks post–tamoxifen induction) and negative sorting of tumoral cells with tumor sorting kit (Miltenyi). For TDCLs generation, KP-sgCtl or KP-sgPink1 TDCLs were made from lung tumors at 10 to 11 weeks post–tumor induction by intratracheal injection of pSECC-sgCtl and pSECC-sgPink1 lentiviral particles, respectively.

KP cell line was cultured in RPMI 1640 medium with Glutamax-I supplemented with 10% fetal bovine serum (FBS), penicillin (100 U/ml), streptomycin (100 ng/ml), and 1 mM sodium pyruvate. The human NSCLC cell line A549 (American Type Culture Collection, CCL-185), wild-type (WT) and PINK1^KO^ MEFs (gift from S. Tait), and LLC1 (ECACC 90020104) cell line were cultured in Dulbecco’s modified Eagle’s medium (DMEM) high glucose (4.5 g/liter) with Glutamax-I supplemented with 10% heat-inactivated FBS, penicillin (100 U/ml), and streptomycin (100 ng/ml).

All cell lines were cultured at 37°C in a humidified atmosphere containing 5% carbon dioxide. Cell line cultures were periodically tested for mycoplasma contamination by polymerase chain reaction (PCR).

For mitophagy induction, KP cells were treated with a mix of 5 μM oligomycin (#75351, Sigma-Aldrich) and 4 μM antimycin A (#A8674, Sigma-Aldrich). MEFs, A549, and TDCLs from KP Lenti-Cre intratracheal model were treated with 1 μM oligomycin and 1 μM antimycin A.

### Isolation and culture of pMEFs

pMEFs were prepared from 13.5 day embryos. Pregnant females were euthanized by cervical dislocation, and uteri were aseptically dissected and transferred to ice-cold phosphate-buffered saline (PBS). Embryos were separated from extraembryonic membranes, rinsed in PBS, and decapitated to collect tissue for genotyping. The liver was removed, and the remaining embryonic tissue was finely minced using sterile surgical scissors in 0.05% trypsin-EDTA (Gibco). Tissues were incubated in trypsin-EDTA for 15 min at 37°C with occasional gentle pipetting to aid dissociation. Enzymatic digestion was terminated by adding an equal volume of pMEF culture medium consisting of DMEM with Glutamax-I (high glucose; Gibco) supplemented with 10% heat-inactivated FBS, 1 mM sodium pyruvate, and penicillin-streptomycin (100 U/ml). The cell suspension was filtered through a 70-μm nylon mesh to remove undigested tissue and centrifuged at 400*g* for 5 min, and the resulting cell pellet was resuspended in fresh pMEF medium.

Cells were plated into 10-cm plate and incubated at 37°C in a humidified atmosphere of 5% CO_2_. After 24 hours, nonadherent debris was removed by replacing the medium. MEFs were expanded for two passages and either used directly or cryopreserved in freezing medium at −150°C for later use.

### sgRNA sequences, nanoblades production, and generation of genetically modified cell lines

Nanoblades were produced as described previously (*45*). Briefly, transfected producer 293T cells were plated at 2.5 × 10^6^ cells/10-cm plate, 24 hours before transfection with the calcium phosphate procedure. Plasmids encoding the GagMLV-CAS9 fusion (1.7 μg), Gag-POLMLV (2.8 μg), guide RNA–expressing plasmid(s) Crizi (4.4 μg), vesicular stomatitis virus glycoprotein (VSVG) (0.4 μg), and the baboon endogenous retrovirus Rless glycoprotein (BaEVRless) (0.7 μg) were cotransfected; medium was replaced 24 hours after transfection by 6 ml of Optimem (Gibco) supplemented with penicillin-streptomycin and Hepes; and supernatants were collected from producer cells after 48 hours. Nanoblade-containing medium was clarified by a short centrifugation (500*g*, 5 min) and filtered through a 0.45-μm pore-size filter before centrifugation (3000*g*, 18 hours, 4°C). The sgRNA sequences used were for mPink1, 5′-CAAGCGCGTGTCTGACCCAC-3′; for unrelevant sequence (Ctl), 5′-CGCGATAGCGCGAATATAC-3′; and for hPink1, 5′-GACCCGTTGGACACGAGACG-3’.

Transductions with nanoblades were performed in 24-well plate seeded with 20.10^4^ cells, in 400 μl of medium to optimize cell/particles interactions for at least 18 hours with polybrene (8 μg/ml), before supplementing with fresh medium. This step was repeated twice to improve editing efficiency. Polybrene was used at a final concentration of 8 μg/ml in the transduction medium. Protein expression of the corresponding targeted gene was evaluated 1 week after the last transduction by Western blot.

### pSECC lentiviral particles production and titration

Lentiviral particles made from pSECC vector (a gift from T. Jacks, MIT) were produced by cotransfection of the lentiviral backbone constructs and packaging plasmids psPAX2 (Addgene, 12260) and VSVG–encoding plasmid phCMV-VSVG (gift from E. Verhoeyen) in 293T cells. Viral supernatants were collected 48 and 72 hours posttransfection and filtered through a 0.45-μm pore-size filter before centrifugation (3000*g*, 18 hours, 4°C). All viral preparations were made in-house, aliquoted, and stored at −80°C. For titration of lentiviral particles for mouse experiments, pSECC preps were functionally tittered by transduction of a reporter line [Green-Go Cre-reporter cell line (*26*)], which turns on expression of GFP upon Cre-mediated recombination and allows quantitation of functional titers derived from the percent of GFP^+^ cells quantified by flow cytometry.

### 2D clonogenic assay

pMEFs from KP-QC mice were infected with either lentiviral particles containing pSECC-sgCtl or pSECC-sgPink1 vectors at a multiplicity of infection (MOI) of 0.5. At 72 to 96 hours posttransduction, the cells were plated in 12-well plate at a density of 500 to 1000 cells per well. Five to ten days later, the cells were washed once with DPBS and stained with crystal violet for 1 hours at room temperature. Last, colonies were washed three times with tap water and air dried. Plates were imaged with IBright FL1500 (Invitrogen), and colony area was determined using colony area plugin (*46*) on ImageJ software.

KP-sgCtl and KP-sgPink1 were plated in 12-well plate at a density of 2000 cells per well and treated with the indicated concentrations of MRTX1133 for 5 to 6 days. The staining was performed as described above.

### NAC assay on pMEFs

pMEFs from KP-QC mice were infected with lentiviral particles containing pSECC-sgCtl vector at an MOI of 0.5. At 96 hours posttransduction, the cells were plated and treated 24 hours later with 4 mM *N*-acetyl-l-cysteine [(pH 7.4) NAC, Sigma-Aldrich, ref. A9165]. Three days after treatment, the cells were lysed and protein extracts were processed for Western blots.

### Cell proliferation assay of KP cell lines

A total of 150,000 KP-sgCtl and KP-sgPink1 cells were plated in six-well plates, and cell proliferation was determined over time until 96 hours. For *KRAS* inhibitor treatment, 7500 KP-sgCtl and KP-sgPink1 cells were plated in 24-well plate. Twenty-four hours postseeding, cells were treated with either Sotorasib or MRTX1133 for 48 hours. Cell count was calculated by 4′,6-diamidino-2-phenylindole (DAPI) staining by flow cytometry on a MacsQuant VYB cytometer.

### Immunofluorescence and confocal microscopy on cell line

Following the indicated treatments, cells were fixed for 15 min in fixation buffer [3.7% formaldehyde in PBS supplemented with 200 mM Hepes (pH7.4)] and washed three times with PBS (Gibco, 14190-094). Cells were permeabilized for 3 min in permeabilization buffer (PBS containing 0.1% Triton X-100), washed three times with PBS, and blocked for 30 min at room temperature in blocking buffer [PBS supplemented with 3% bovine serum albumin (BSA)]. Cells were then incubated overnight at 4°C in a humidified chamber protected from light with primary antibodies at the appropriate dilutions.

The following day, the cells were washed three times with PBS and incubated for 1 hour at room temperature in the dark with appropriate fluorescent-conjugated secondary antibody (Molecular Probes) and DAPI (Sigma-Aldrich, D9542) in blocking buffer. After three additional washes with PBS, the samples were mounted with Fluoromount-G (Life Technologies, 00-4958-02).

Confocal images were acquired using a Nikon A1R confocal microscope equipped with a Nikon Plan Fluor HN2 60× oil immersion objective. A digital zoom factor of 3 and an averaging setting of 3 were applied for representative images acquisition.

### Mice sample collection

Mice were euthanized by cervical dislocation, and lungs were rapidly excised. Tissues were either snap-frozen in liquid nitrogen and stored at −80°C for subsequent biochemical analysis or fixed overnight by immersion in freshly prepared, 0.22 μm–filtered fixative consisting of 3.7% formaldehyde (Sigma-Aldrich, 252549), 200 mM Hepes (pH 7.0), and 30% sucrose (Sigma-Aldrich, 55016) in PBS (Gibco, 14190-094).

The following day, fixed tissues were washed in PBS and either dehydrated in 70% ethanol at 4°C for paraffin embedding or incubated for 2 hours at room temperature in the dark in optimal cutting temperature (OCT) compound (Tissue-Tek, Sakura, 4583), embedded, and stored at −80°C until further processing. Frozen lung samples were homogenized using a stainless steel tissue grinder (BioSpec Products, 1292) and subsequently processed for either protein or total RNA extraction.

### Tissue sectioning

OCT-embedded lungs were cryosectioned at a thickness of 8 μm using a Leica CM1950 cryostat equipped with disposable microtome blades (MW35 Ultra, 3053835). Sections were mounted onto Superfrost Plus adhesion slides (Epredia, J7800AMNZ), air-dried, and stored at −80°C in sealed slide boxes until further use.

Formalin-fixed paraffin-embedded (FFPE) tissues were sectioned at a thickness of 5 μm using an HM340E microtome and mounted onto Superfrost Plus adhesion slides (Epredia, J7800AMNZ). Sections were stored at room temperature in the dark until further processing.

### Immunofluorescence on tissue sections

Lung cryosections were thawed for 5 min at room temperature, rehydrated in 1× PBS, and blocked/permeabilized for 1 hour in 1× PBS containing 5% BSA and 0.3% Triton X-100. Sections were then incubated overnight at 4°C in a humidified chamber protected from light with primary antibodies (Table 1) diluted in 1× PBS supplemented with 3% BSA and 0.1% Triton X-100. The following day, the slides were washed three times with 1× PBS and incubated for 1 hour at room temperature with Alexa Fluor–conjugated secondary antibodies and DAPI (Sigma-Aldrich, D9542) in antibody dilution buffer. After three washes with 1× PBS, sections were mounted with Fluoromount-G (Life Technologies, 00-4958-02).

**Table 1.** Antibodies.

Antibody	Application	Supplier	Reference
PINK1 (clone EPR20730)	For human sample	Abcam	Ab216144
PINK1	IP	MRC-PPU	S774C
PINK1	Western blot (mouse sample)	MRC-PPU	S086D
PINK1	Western blot (human sample)	Cell Signaling Technology	6946
P-Ser^65^ ubiquitin	Western blot	Cell Signaling Technology	70973
P-Ser^65^-ubiquitin (clone E2J6T)	IF and IHC-DAB	Cell Signaling Technology	62802
P-ERK	IHC-DAB	Cell Signaling Technology	4376
	Western blot		
Ki67	IHC-DAB	Abcam	Ab15580
CC3	IHC-DAB	Cell Signaling Technology	9661
CD45 (D3F8Q)	IF	Cell Signaling Technology	70257
COX IV	IF	ProteinTech	11242–1-AP
P-Ser^345^ CHK1	Western blot (mouse sample)	Cell Signaling Technology	2348
CHK1	Western blot (mouse sample)	Cell Signaling Technology	2360
NRF2	Western blot (mouse sample)	Cell Signaling Technology	12721
γH2AX	Western blot (mouse sample)	Abcam	ab81299
ATG5	Western blot (mouse sample)	Cell Signaling Technology	12994
ATG7	Western blot (mouse sample)	Cell Signaling Technology	8558
LC3	Western blot (mouse sample)	Novus Biologicals	NB100-2220
E-cadherin–AF647	IF	Biolegend	BD147307
E-cadherin	IHC-DAB	Cell Signaling Technology	3195
ACTIN	Western blot	Santa Cruz Biotechnology	SC47778
P-Ser^111^–RAB8A	Western blot	Abcam	Ab267492
RAB8A	Western blot	Proteintech	55296-1-AP
CC10	IHC-DAB	Proteintech	10490-1-AP
RAS G12D	Western blot	Cell Signaling Technology	144295
RAS	Western blot	Proteintech	12063-1-AP
ERK2	Western blot	Santa Cruz Biotechnlogy	SC1647
P-Ser^616^ DRP1	Western blot	Cell Signaling Technology	4494
DRP1	Western blot	Cell Signaling Technology	8570
Anti-rabbit IgG HRP linked	Western blot	Cell Signaling Technology	7074
Anti-mouse IgG HRP linked	Western blot	Cell Signaling Technology	7076
Rabbit anti-sheep IgG (H + L) secondary antibody, HRP	Western blot	Invitrogen	31480
Donkey anti-rabbit Alexa Fluor 568	IF	Invitrogen	A10042
Donkey anti-rabbit IgG (H + L) highly cross-adsorbed secondary antibody, Alexa Fluor 647	IF	Invitrogen	A-31573

Confocal images were acquired on a single focal plane using a Nikon A1R confocal microscope equipped with a Nikon Plan Fluor HN2 40× oil immersion objective. COX IV fluorescence intensity was quantified using Qupath Software after manual annotation of ROIs (bronchi, hyperplasia, and tumor) and mean gray intensity values from the COX IV channel were used for statistical analysis.

FFPE tissue slides were incubated at 60°C for 30 min, deparaffinized in xylene, rehydrated in deionized water, and subjected to antigen retrieval in citrate buffer (pH 6.0). Sections were blocked/permeabilized for 1 hour in PBS containing 5% BSA and 0.3% Triton X-100 before overnight incubation at 4°C with anti-CD45 primary antibody (Table 1) diluted in PBS supplemented with 3% BSA and 0.1% Triton X-100. Slides were subsequently washed and incubated for 1 hour at room temperature with Alexa Fluor–conjugated secondary antibodies and DAPI, and mounted with Fluoromount-G.

Whole-slide fluorescent images were acquired using a Hamamatsu NanoZoomer 2.0-HT scanner and analyzed with Qupath Software. Cell detection was performed on the DAPI channel with a 5-μm cell expansion to define the cytoplasmic compartment, and a single “Cell: Channel mean” intensity threshold was applied to each sample. CD45-positive cells were identified on the basis of the proportion of CD45-positive pixels within the corresponding cell area and quantified in five randomly selected ROI per mouse.

### Immunohistochemistry on FFPE lung tissue sections

Following sectioning, FFPE slides were incubated at 60°C for 30 min, deparaffinized in xylene, rehydrated in deionized water, and subjected to antigen retrieval in either tris-EDTA buffer (pH 9.0) or citrate buffer (pH 6.0), depending on the primary antibody. Endogenous peroxidase activity was quenched with 0.3% hydrogen peroxide for 10 min, and sections were blocked for 1 hour at room temperature in PBS containing 3% BSA. Primary antibodies (Table 1) were incubated for 90 min or overnight at 4°C. Sections were subsequently incubated with biotinylated goat anti-rabbit secondary antibody for 30 min, followed by streptavidin-peroxidase for 10 min. Immunoreactivity was visualized using a 3,3′-diaminobenzidine (DAB) detection kit (Abcam, ab64261), and sections were counterstained with hematoxylin. Whole-slide images were acquired using a Hamamatsu NanoZoomer 2.0-HT scanner and analyzed with Qupath Software.

Cell detection was performed on the Optical density sum image, followed by a 5-μm cell expansion to define the cytoplasmic compartment. For each antibody, a single intensity threshold was applied to either the cell: DAB OD mean or nucleus: DAB OD mean parameter, as appropriate. Positive cells were identified on the basis of the proportion of DAB-positive pixels within the corresponding cell area and quantified in the indicated ROIs.

### mito-QC analysis

Tissue cryosections were thawed at room temperature, washed in PBS, incubated with DAPI, and mounted with Fluoromount-G (Life Technologies, #00-4958-02). Images were acquired using a Nikon A1R confocal microscope equipped with a Nikon Plan Fluor HN2 60× oil immersion objective. Representative images were collected at 60× magnification with an averaging setting of 4 and a digital zoom factor of 2.

Mitophagy was quantified using the ImageJ plugin mito-QC_counter (*23*) with the following parameters: image smoothing radius = 1 and red-to-green fluorescence ratio threshold = 1.5 to 2. For both in vivo models (tamoxifen-induced and Lenti-Cre), two to three regions were analyzed in normal adjacent tissue and lesional tissue (hyperplasia or adenocarcinoma), and two to eight ROIs were quantified per region. Results are expressed as the number of mitolysosomes per square micrometer.

pMEF PQC, KQC, and KPQC, transduced with pSECC-sgCtl lentiviral particles, were plated and treated for 4 hours with Pepstatin (10 μg/ml; Enzo Life Sciences, ALX-260-085) and E64 (APExBIO, ref. A2576). Cells were then fixed for 15 min in fixation buffer (3.7% formaldehyde in 1× PBS supplemented with 200 mM Hepes) and washed three times with 1× PBS.

Confocal images were acquired using a Nikon A1R microscope equipped with a Nikon Plan Fluor HN2 40× oil immersion objective with an averaging setting of 4. Representative images were collected at 60× magnification using an averaging setting of 4 and a digital zoom factor of 2. Mitophagy was quantified using the ImageJ plugin mito-QC_counter with the same analysis parameters as described above (image smoothing radius = 1; red-to-green fluorescence ratio threshold = 1.5 to 2). For each genotype (PQC, KQC, and KPQC), three randomly selected fields were imaged, and three to six cells per field were analyzed. Results are expressed as the number of mitolysosomes per cell.

### Mitochondrial network fragmentation analysis

pMEFs isolated from KP-QC mice were transduced with lentiviral particles carrying the pSECC-sgCtl vector at an MOI ranging of 0.5. Four days after transduction, the cells were seeded into 24-well plates at a density of 15,000 cells per well. Five days later, the cells were fixed for 15 min in fixation buffer (3.7% formaldehyde in 1× PBS supplemented with 200 mM Hepes) and washed three times with 1× PBS.

Mitochondrial network fragmentation was quantified using the ImageJ Analyze Skeleton plugin. Only CRE-positive cells were included in the analysis, and image thresholding was performed on the GFP channel of the mito-QC reporter. The “Prune ends” option was enabled to eliminate end points. A fragmentation index was then calculated as the ratio of the number of end points to the total number of branches.

### Cell death assay on FFPE tumor sections

Following sectioning, FFPE tissue slides were incubated at 60°C for 30 min and deparaffinized in xylene for 30 min. After rehydration in deionized water for 5 min, antigen retrieval was performed by boiling the slides in citrate buffer (pH 6.0). Sections were subsequently incubated with equilibration buffer and TUNEL reaction mixture according to the manufacturer’s instructions (DeadEnd Fluorometric TUNEL System, Promega, G3250).

Fluorescent whole-slide images were acquired using a Hamamatsu NanoZoomer 2.0-HT scanner and analyzed with Qupath Software. Cell detection was performed using the DAPI channel, followed by a 5-μm cell expansion to define the cytoplasmic compartment. For each sample, a single intensity threshold was applied to the Cell: Channel mean parameter. Cells were classified as TUNEL-positive based on the proportion of TUNEL-positive pixels within the corresponding cell area.

### PINK1 immunoprecipitation

Cells or tissue powder were lysed in ice-cold lysis buffer containing the following: 50 mM tris/HCl (pH 7.5), 1 mM EDTA (pH 8.0), 1 mM EGTA (pH 8.0), 1% Triton X-100, 0.25 M sucrose, 150 mM NaCl, 2 mM sodium orthovanadate, 1 mM NaF, 10 mM glycerophosphate, 1 mM dithiothreitol, 2 mM phenylmethylsulfonyl fluoride, aprotinin (10 μg/ml), and leupeptin (10 μg/ml). After 15 min on ice, the lysates were clarified by centrifugation at 20,000*g* for 10 min at 4°C. Precleared lysates (1 mg) were incubated with 5 μg of sheep anti-mouse PINK1 (S774C, 3rd Bleed, MRC PPU Reagent) with protein A/G plus sepharose beads for 16 hours at 4°C under end-over-end agitation. Then, the beads were washed four times in ice-cold corresponding lysis buffer. The immunoprecipitated protein complexes were then resuspended in Laemmli buffer and boiled for 5 min at 95°C before immunoblot analysis.

### Western blot analysis

Whole-cell protein extracts were obtained by lysing cells or tissue powder with Laemmli buffer [2% SDS, 80 mM tris (pH 6.8), and 10% glycerol]. Protein concentration was measured with the BCA Protein Assay Reagent kit (Pierce). Twenty to forty micrograms of protein extracts were subjected to reducing conditions, loaded onto a polyacrylamide gel, and then transferred to Immobilon-P membranes from Millipore. One hour after blocking the nonspecific binding sites with 5% (w/v) nonfat milk or 5% BSA in tris-buffered saline, membranes were incubated overnight at 4°C with the following specific primary antibodies (see Table 1). Antibody binding was detected using a secondary antibody conjugated to horseradish peroxidase and the enhanced chemiluminescence detection system (Amersham, Little Chalfont, UK). Quantification of band intensities was conducted using Fiji software. Relative density of each protein and condition was referred to the internal normalization control.

### RNA extraction and RT-qPCR

Total RNA was isolated from either cell lines or lung tissue powder using TRIzol reagent (Invitrogen) according to the manufacturer’s instructions. The RNA concentration was assessed using a Nanodrop spectrophotometer (Thermo Fisher Scientific) by measuring the absorbance at 260 and 280 nm. Reverse transcription was performed on 1 μg of RNA using the Omniscript RT Kit (Qiagen, #205113).

The relative mRNA levels of murine specific mRNA were obtained by real-time quantitative (qPCR) on the StepOne Real-Time PCR System (Thermo Fisher Scientific) using either TaqMan assays (mouse Rplp0 ID Mm00725448_s1, mouse Pink1 ID Mm00550827_m1) and SYBR Green (Applied Biosystems, 4367659) according to the manufacturer’s instructions. The following primers were used with SYBR Green chemistry: mouse Rplp0 Forward: 5′-GCTTCGTGTTCACCAAGGAGGA-3′, Reverse: 5′-GTCCTAGACCAGTGTTCTGAGC-3′ and mouse Trp53 Forward: 5′-CACAAAAACAGGTTAAACCCAG-3′, Reverse: 5′-AGCACATAGGAGGCAGAGAC-3′. The relative mRNA levels were normalized to mouse Rplp0.

### siRNA reverse transfection

A total of 40,000 pMEFs (12-well plate) or 100,000 KP (6-well plate) cells were reverse-transfected with siRNA targeting either mouse Pink1, Atg5, or Atg7. Gene silencing was achieved using a pool of esiRNA targeting Pink1 (MISSION esiRNA, Sigma-Aldrich, EMU074421) or duplex DsiRNA (Integrated DNA Technologies) targeting Pink1 (ID mm.Ri.Pink1.13.1), Atg5 (IDs mm.Ri.Atg5.13.1, mm.RiAtg5.13.3), and ATG7 (IDs mm.Ri.Atg7.13.1, mm.Ri.Atg7.13.2). A nontargeting negative control DsiRNA (Integrated DNA Technologies, 51-01-14-03) was also used. Transfections were performed using Lipofectamine RNAiMAX (Thermo Fisher Scientific, 13778075) according to the manufacturer’s instructions. Briefly, siRNA (25 nM final concentration) and Lipofectamine RNAiMAX were diluted separately in Opti-MEM reduced serum medium, incubated for 10 to 20 min at room temperature to allow complex formation, and then cells were added onto the complex. For pMEFs, 24 hours later, the medium was refreshed and cells were infected with either irrelevent or pSECC-sgCtl lentiviral vectors in the presence of polybrene (8 μg/ml) and further processed for 2D clonogenic assays. For KP cells, cells were trypsinized 48 hours posttransfection and seeded for 2D clonogenic assay. Gene knockdown was assessed by Western blot.

### Intratracheal injection

Tumors were induced by intratracheal injection of lentiviral particles (between 20,000 and 50,000 transforming units in 75 μl) expressing Cre recombinase and Cas9-sgRNA (pSECC vector) as previously described (*26*, *47*) in mice that were between 8 and 12 weeks of age.

### Mouse breeding and maintenance

All animal procedures were performed according to the guidelines of the Institutional Animal Care and Use Committee and the regional ethics committee (approval references PEA-673). All mice strains used are on C57Bl6 background: LSL-*KRAS*G12D/+ (B6.129S4-*KRAS*tm4Tyj/J) mice carry a Lox-Stop-Lox (LSL) sequence with the *KRAS*G12D point mutation, which can be excised by Cre-mediated recombination. CCSP-CreER/^+^ (B6N.129S6(Cg)^−^ Scgb1a1tm1(cre/ERT)Blh/J) mice contained an inducible Cre recombinase inserted into the CCSP locus. p53^fl/fl^ (FVB.129P2-*Trp53^tm1Brn^*/Nci) contained LoxP sites inserted into intron 1 and intron 10 of the p53 locus. Mito-QC mitophagy reporter mice were obtained from I. Ganley (Dundee, Scotland) (*22*). Briefly, targeted transgenesis was used to achieve a constitutive knock-in of a fusion protein combining a pH-insensitive fluorescent marker (mCherry), a pH-sensitive fluorescent marker (GFP), and the mitochondrial targeting sequence of FIS1 (mCherry-GFP-FIS1^101–152^) into the Rosa26 locus.

We generate three different mice models: (i) The CK-QC mice model consists of the breeding of CCSP-CreER/+ (C) mice with LSL-*KRAS*G12D/+ (K) and mito-QC (QC) mice; (ii) the CKP-QC mice model consist of same previous breeding with p53^fl/fl^ (P) mice; (iii) the KP-QC mice resulting from the breeding of LSL-*KRAS*G12D/+ (K) mice with p53^fl/fl^ (P) mice and mito-QC (QC).

For each experiment, cohoused littermate controls were used. All experiments used a mixture of female and male mice. Mice were bred and housed at C3M on a 12-/12-hour light/dark cycle with ad libitum access to food and water, in constant room temperature (22°C).

### Allograft experiments and tumor growth follow-up

Eight-week-old WT syngeneic C57BL/6 mice, purchased from Janvier, were subcutaneously injected with 2.10^6^ of KP sgCtl or KP sgPink1. Mice were inspected daily for tumor development and increase in tumor size was measured with a caliper. Tumor volume was calculated as follows: (length × width × height)/2. For allograft experiment using MRTX1133 inhibitor, once tumor volume reached 100 mm3, mice were treated daily IP with MRTX1133 (3 mg/kg) or vehicle [10% Captisol, 50 mM citrate buffer (pH 5)].

### Tamoxifen induction

CK-QC (K model) and CKP-QC (KP model) mice were intraperitoneally injected with 200 μl of tamoxifen (Sigma-Aldrich) solution (20 mg/ml in corn oil) at 8 to 10 weeks of age to induce lung carcinogenesis.

### Integrative RNA-seq analysis from lung cancer GEMM

Integrative RNA-seq analysis was performed using published datasets on GEO portal from lung cancer GEMM harboring *KRAS*^G12D^ mutation in the absence or presence of Trp53 via either Adeno-Cre or Lenti-Cre instillation. Pretreatment based on common gene symbols among datasets was done, leading to a total number of 14,622 genes. We defined two groups, WT group (GSE171217, GSE70046, and GSE139347; total number of 7 samples) and tumor group (GSE171217, GSE94927, GSE52594, GSE137396, GSE158110, and GSE161609; total number of 32 samples) The differential expression analysis was conducted using the limma package in R, applying the thresholds |Fold Change| ≥ 1 and FDR < 0.05, which identified 3081 differentially regulated genes.

Gene Set Enrichment Analysis (GSEA) was then performed in preranked mode using the ranked gene list derived from the limma statistics. GSEA results were represented as a bubble plot, displaying normalized enrichment score and FDR for the enriched pathways.

### Human LUAD TCGA RNA-seq analysis

Gene set enrichment was performed using the GSVA R package in single-sample GSEA (ssGSEA) mode on the normalized expression matrix. The Reactome Pathways collection was used as the reference gene set database. ssGSEA scores were computed for each sample to quantify the relative activation level of each pathway, with normalization enabled to ensure comparability across samples. Differential pathway activity between tumor and normal samples was assessed using the Limma linear modeling framework. Pathways with an adjusted FDR < 0.05 (Benjamini-Hochberg correction) were considered significantly enriched. Heatmaps (blue-white-red color scale) were generated to display pathway enrichment and clustering patterns between tumor and normal samples.

ssGSEAscores for REACTOME_MITOPHAGY (Tomm40, Ubc, Vdac3, Sqstm1, Ubb, Tbk1, Vdac1, Rps27a, Mterf3, Vdac2, Tomm22, Tomm70a, Prkn, Csnk2b, Fundc1, Optn, Map1lc3a, Src, Mfn1, Pink1, Tomm7, Mfn2, Pgam5, Ulk1, Map1lc3b, Atg12, Atg9a, Tomm6, Atg5, Ube2l3, Csnk2a2, Uba52rt, Csnk2a1, Ube2n, Ube2d3, Tomm5, Ube2v1, Uba52, Ube2d2a, and Tomm20) were compared between the tumor and normal groups using the Wilcoxon rank-sum test, and results were visualized with boxplots annotated with adjusted *P* values.

### (Phospho)proteomic dataset from cohorts of patients with LUAD

(Phospho)proteomic dataset from LUAD cohorts were analyzed on National Institutes of Health (NIH) Proteomic Data Common portal (https://pdc.cancer.gov/pdc/). The following studies were used to look for mitophagy-related readouts: Academia Sinica LUAD100 (*48*), ID PDC000219 (proteomic, *n* = 186 replicates, 93 patients with nonlesional and tumor samples) and ID PDC000220 (phosphoproteomic, *n* = 37 replicates, 23 patients with nonlesional and tumor samples); CPTAC LUAD Discovery (*49*), ID PDC000153 (proteomic, *n* = 101 nonlesional and *n* = 112 tumor samples) and ID PDC000149 (phosphoproteomic, *n* = 28 nonlesional and *n* = 32 tumor samples). Only qualified and LUAD-related samples were used in the analysis. Data were represented as Log_2_Ratio.

### Cohort of patients with LUAD

In total, 32 patients with NSCLC diagnosed from 2018 to 2023 were retrospectively included in this study at the Laboratory of Clinical and Experimental Pathology (Pasteur Hospital, Nice, France; ISO 15189). All samples were collected from patients with LUADs. The median age at diagnosis was 70 years old (range 41 to 89 years) with 21 males and 11 females’ patients. All patients were former (*9*) or active (*23*) smokers. The tumors were classified on surgically resected specimens, according to the 2021 World Health Organization Classification of Thoracic Tumors (Journal of Thoracic Oncology volume 17, issue 3, March 2022, pages 362 to 387). Most (29 tumors) were early stages NSCLC (IA to IIIA) and three cases were advances cases (IV). The time of fixation of all the specimens was controlled. Biopsies were fixed from 6 to 12 hours and surgically resected specimens (approximatively 1 cm^2^) were fixed from 12 to 36 hours in 10% formalin. The number of biopsies ranged between 3 and 8 (average, 5.5). The study was performed in accordance with the guidelines of the declaration of Helsinki, approved by the local ethics committee (ref 2024: 1031, Nice University Hospital) and all patients provided written informed consent. Immunohistochemistry was carried out on 4-μm-thick FFPE tissue sections using the anti–P-Ser^65^–ubiquitin (clone E2J6T) and anti-PINK1 (clone EPR20730) antibodies. Staining procedures were performed on a Ventana BenchMark Ultra automated immunostainer (Tuczon, AZ, USA). The OptiView DAB IHC Detection Kit (Ventana) and the OptiView Amplification Kit (Ventana) were used according to the manufacturer’s recommendations. Sections were counterstained with hematoxylin and bluing reagent and coverslipped. IHC stainings were classified according to the tumor cell intensity as 0, no staining; +1, faintly stained tumor cells; +2, moderately stained; and + 3, strongly cytoplasmic stained tumor cells.

### Mouse scRNA-seq analysis (GSE152607)

Mitophagy signature was evaluated in processed mouse scRNA-seq dataset (GSE152607) coming from K(P) lung cancer GEMM by adenoCre administration. A total of 3891 tumoral cells were analyzed using interactive interface on the Broad Single Cell Portal (https://singlecell.broadinstitute.org/single_cell/study/SCP971/emergence-of-a-high-plasticity-cell-state-during-lung-cancer-evolution-timecourse-smartseq2). The following gene set was applied (Fkbp8, Tomm22, Bcl2l13, Fundc1, Atg12, Tax1bp1, Tomm20, Mul1, Vdac2, Bnip3, Vdac3, Tomm40, Atg7, Tomm70a, Fis1, Vdac1, Tomm5, Csnk2b, Atg5, Bnip3l, Csnk2a1, Nbr1, Dnml1, Uba52, Ambra1, Tomm7, Optn, Pink1, and Sqstm1). Gene signature collapse by mean was applied to generate tSNE representation of mitophagy signature across time points. Data were further exported to Phantasus portal for analysis. Calculated annotation was created by displaying the mean for each gene of the signature. Then, a cell collapse was done for each group, and Limma2 differential analysis was performed to compare group 1 (early and K samples) and group 2 (KP samples). Adjusted *P* value was displayed for each single gene.

### Tumor burden evaluation

Serial sections of 5 μm of thickness were prepared from both FFPE (HM340E Niagara system) and OCT (CM350 cryostat) embedded samples. Standard hematoxylin and eosin (H&E) staining was performed for tissue morphology observation.

To quantify the tumor burden per mice, H&E-stained sections were scanned at 20× magnification with a Hamamatsu Nanozoomer 2.0HT scanner. After importing scan slides to ImageJ (NIH), one reader counted lesions on three different sections per lung. Individual lesions were manually circled and their areas measured. The sum of all tumor areas was divided by total lung area and plotted as percentage.

### Mito-mKeima analysis by flow cytometry

*KRAS*^G12D/^+^^;Tp53^−/−^ tumor–derived cells (parental, sgCtl, and sgPink1) were infected with mito-Keima–expressing lentiviral vector (*14*). The excitation fluorescence of this mitochondrial targeted fluorescent protein is pH dependent, making it a perfect biosensor of mitochondrial degradation by the lysosome: Excitation at 488 nm and emission at >620 nm were used to detect mitochondria in the cytosol, and excitation at 561 nm and emission at >620 nm were used to detect mitochondria in lysosomes. Mito-Keima was analyzed by flow cytometry using MACSQuant VYB and MACSQuant software (Miltenyi Biotec, Bergisch Gladbach, Germany).

### Statistical methods

Data are expressed as the means ± SEM. Statistical significance was performed using two-tailed parametric Student’s *t* test, one-way analysis of variance (ANOVA) with Tukey’s posttest analysis or two-way ANOVA according to the dataset (GraphPad software, San Diego, CA). Results were considered as statistically significant when *P* < 0.05. All the statistical details of experiments can be found in the figure legends.
