# Supplementary material for: PINK1-mediated mitophagy supports lung cancer initiation and limits the efficacy of KRAS-targeted therapy
Source: Sci Adv. 2026 Oct 16;12(42):eaef8820. doi: 10.1126/sciadv.aef8820 (PMC13674508; doi:10.1126/sciadv.aef8820)
Supplement: Supplementary file 1 [file sciadv.aef8820_sm.pdf]

Science Advances
AAAS

Supplementary Materials for

PINK1-mediated mitophagy supports lung cancer initiation and limits the efficacy of *KRAS*-targeted therapy

Diogo Gonçalves *et al.*

Corresponding author: Sandrine Marchetti, sandrine.marchetti@univ-cotedazur.fr;
Jean-Ehrland Ricci, ricci@unice.fr

Sci. Adv. **12**, eaef8820 (2026)
DOI: 10.1126/sciadv.aef8820

This PDF file includes:

Figs. S1 to S6

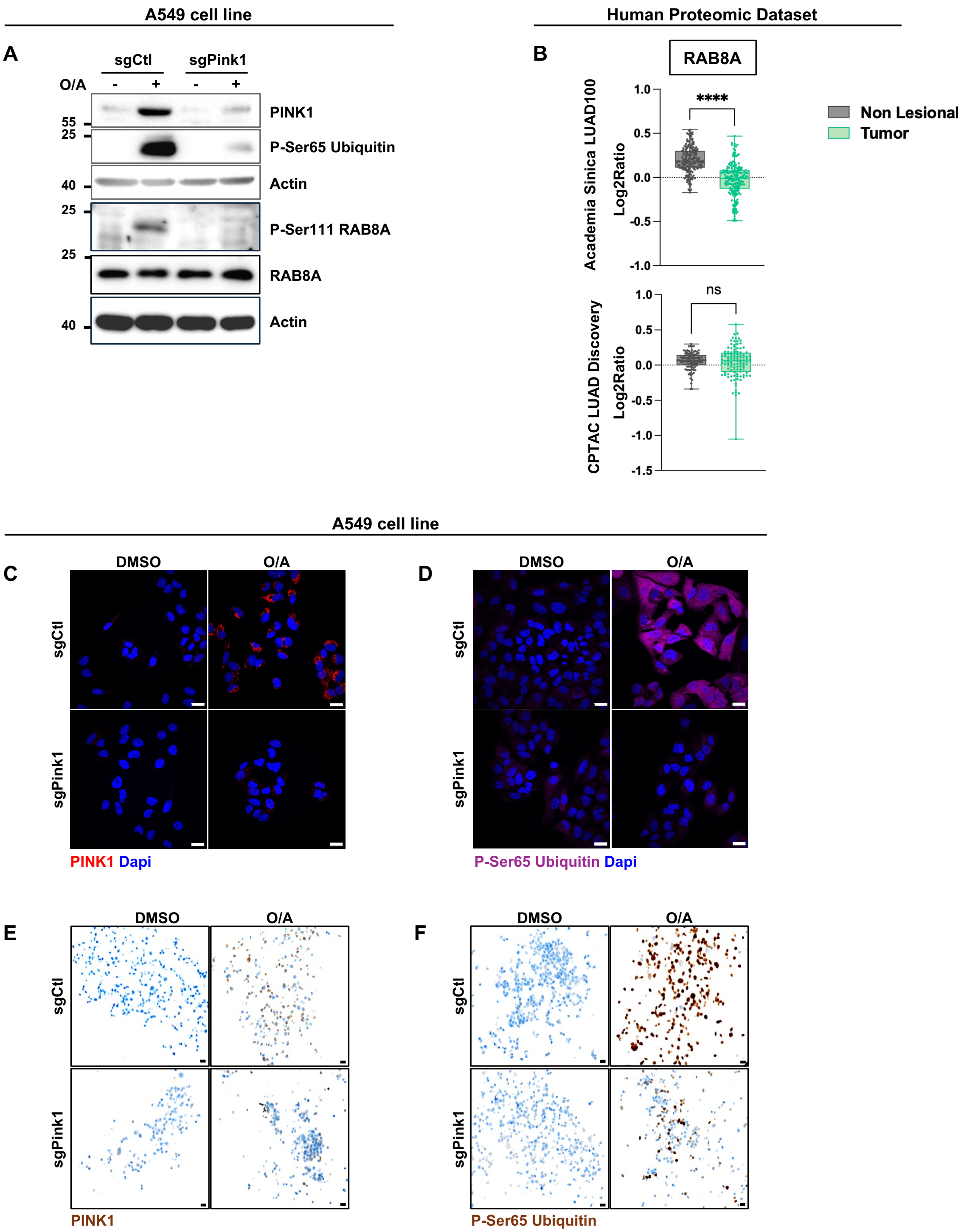

Fig. S1. Validation of antibodies for the detection of PINK1 expression and activity induced during mitophagy in the human lung cancer cell lineA549

(A) Western blot on A549-sgCtl and A549-sgPink1 treated 24h with either vehicle or 1µM oligomycin and 1µM antimycin A. **(B)** RAB8A relative expression between normal adjacent and tumor tissues in published LUAD phosphoproteomic and proteomic databases from the NIH

Protein Data Common (PDC). Data are represented in Log2ratio. Mann-Whitney unpaired t-test. **** P<0.0001. **(C and D)** Confocal images of PINK1 ICC (C) and pUb-Ser65 (D) on A549-sgCtl and A549-sgPink1 treated 24h with either vehicle or 1µM oligomycin and 1µM antimycin. Scale bar 20µm. **(E, F)** PINK1 (E) and P-Ser65-Ubiquitin (F) IHC-DAB on A549-sgCtl and A549-sgPink1 treated 24h with either vehicle or 1µM oligomycin and 1µM antimycin. Photos were taken with a scanner microscope at 20X magnification. Scale bar 20µm.

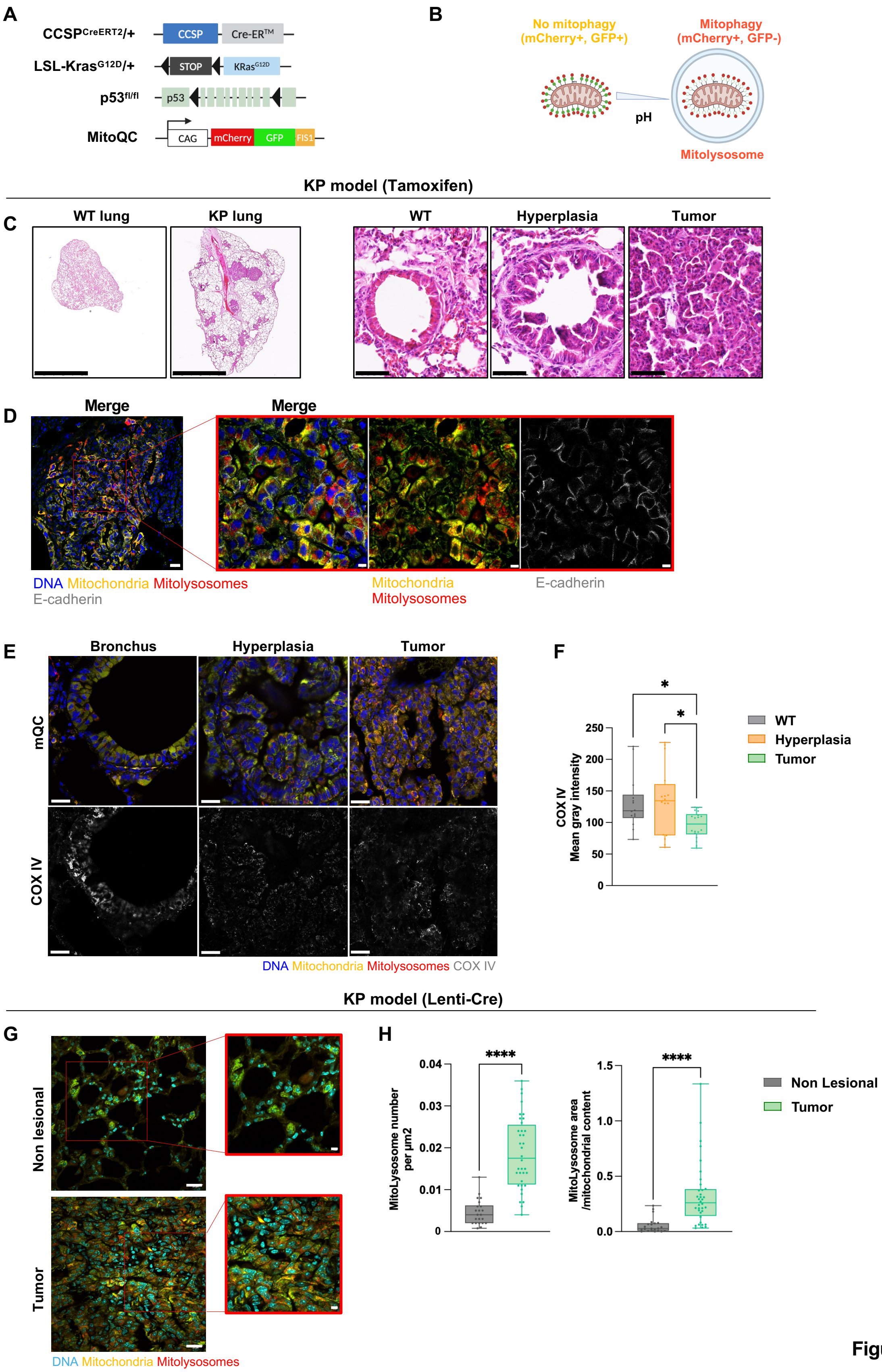

A
CCSPCreERT2/+
CCSP
Cre-ERTM
LSL-KrasG12D/+
STOP
KRasG12D
p53fl/fl
p53
MitoQC
CAG
mCherry
GFP
FIS1
B
No mitophagy
(mCherry+, GFP+)
Mitophagy
(mCherry+, GFP-)
pH
Mitolysosome
KP model (Tamoxifen)
C
WT lung
KP lung
WT
Hyperplasia
Tumor
D
Merge
Merge
DNA Mitochondria Mitolysosomes
E-cadherin
Mitochondria
Mitolysosomes
E-cadherin
E
Bronchus
Hyperplasia
Tumor
mQC
COX IV
DNA Mitochondria Mitolysosomes COX IV
F
COX IV
Mean gray intensity
WT
Hyperplasia
Tumor
KP model (Lenti-Cre)
G
Non lesional
Tumor
DNA Mitochondria Mitolysosomes
H
MitoLysosome number
per µm2
MitoLysosome area
/mitochondrial content
Non Lesional
Tumor

Figure S2

Fig. S2. Mitophagy is mainly activated in tumoral cells during lung cancer progression in mice

(A) Breeding scheme for K-QC and KP-QC *in vivo* mouse model of lung cancer, originated from CCSP positive cells upon Tamoxifen injection (CCSP-Cre-ER). Created in BioRender. Gonçalves, D. (2026) https://BioRender.com/yaxmaob. **(B)** Schematic representation of the mito-QC principle for the monitoring of mitophagy. Under basal condition, both mCherry and GFP are expressed on mitochondria based on FIS1 targeting sequence. Upon mitochondrial stress, mitochondria are targeted to lysosomes, where GFP fluorescence is quenched under acidic environment, resulting in mCherry positive dots by confocal fluorescence microscopy. Created in BioRender. Gonçalves, D. (2026) https://BioRender.com/gxvksdj**.** **(C)** Hematoxylin Eosin staining in WT and KP-QC, 11 weeks after tamoxifen injection. Lung tissues are imaged with a scanner microscope at large scale (1.25X) and 40X magnification. Scale bar 2.5mm for large scale and 50µm for magnification. **(D)** Confocal microscopy of E-cadherin immunostaining on tumor area from KP-QC mouse specimen highlighting that most of the mitolysosomes are located in E-cadherin positive cells (tumoral cells). Scale bar 20µm and 5µm for higher magnification. **(E)** Confocal microscopy of COX IV immunostaining on bronchus, hyperplasia and tumor area from KP-QC mice, 12 weeks post tamoxifen injection. Scale bar 20µm. **(F)** Corresponding quantification of COX IV immunostaining (n=1 mouse per group, 2-3 zones per group, 6 ROI per zones). One-way ANOVA, *adj Pval = 0.0468 and 0.0237 (WT vs Tumor and Hyperplasia vs Tumor respectively). **(G)** Mito-QC visualization on frozen lung sections from KP-QC mice (Lenti-Cre model) 17 weeks post intratracheal injection. Scale bar 20µm and 5µm for higher magnification. **(H)** Corresponding quantification of mitolysosomes number and size from non-lesional and lesional areas (n=3 KPQC mice, 1-3 zones per mouse, 1-6 ROI per zone). Mann-Whitney unpaired t-test, ****P <0.0001.

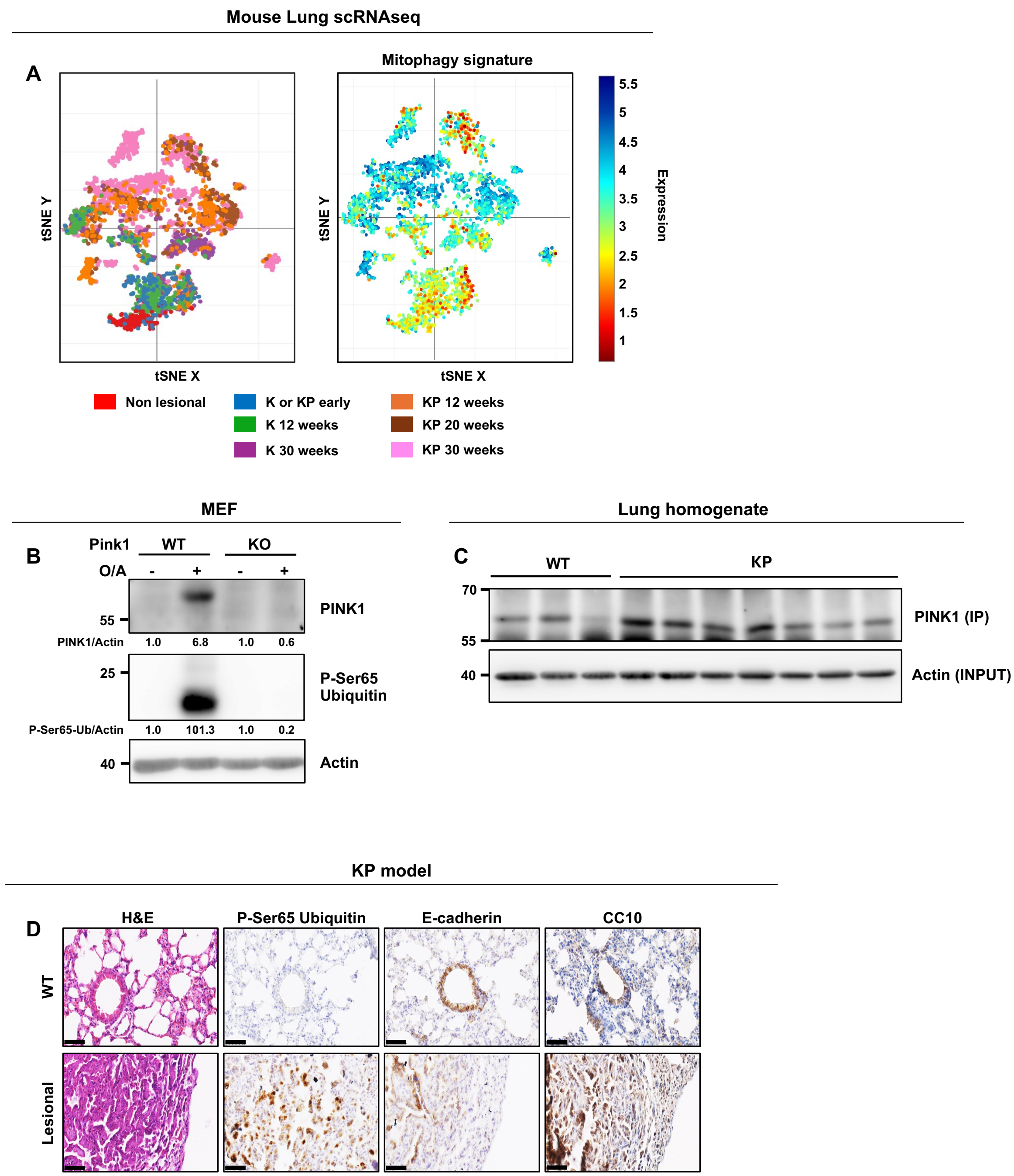

Fig. S3. PINK1-dependent mitophagy is activated during lung cancer progression in mice.

(A) tSNE representation of published lung cancer GEMM scRNAseq (GSE152607) and corresponding mitophagy signature by collapsed mean expression. **(B)** Western blot of WT and PINK1 KO MEF treated with either vehicle or oligomycin and antimycin A (1µM each) for 24h. **(C)** PINK1 immunoprecipitation on enriched lesions from KP-QC mice, 12 post Tamoxifen administration, as in figure 3B. **(D)** Hematoxylin Eosin and IHC staining of P-Ser65 Ubiquitin, E-cadherin and CC10 on FFPE lung sections from WT and KP-QC mice 13 weeks post Tamoxifen injection. Scale bar 100µm.

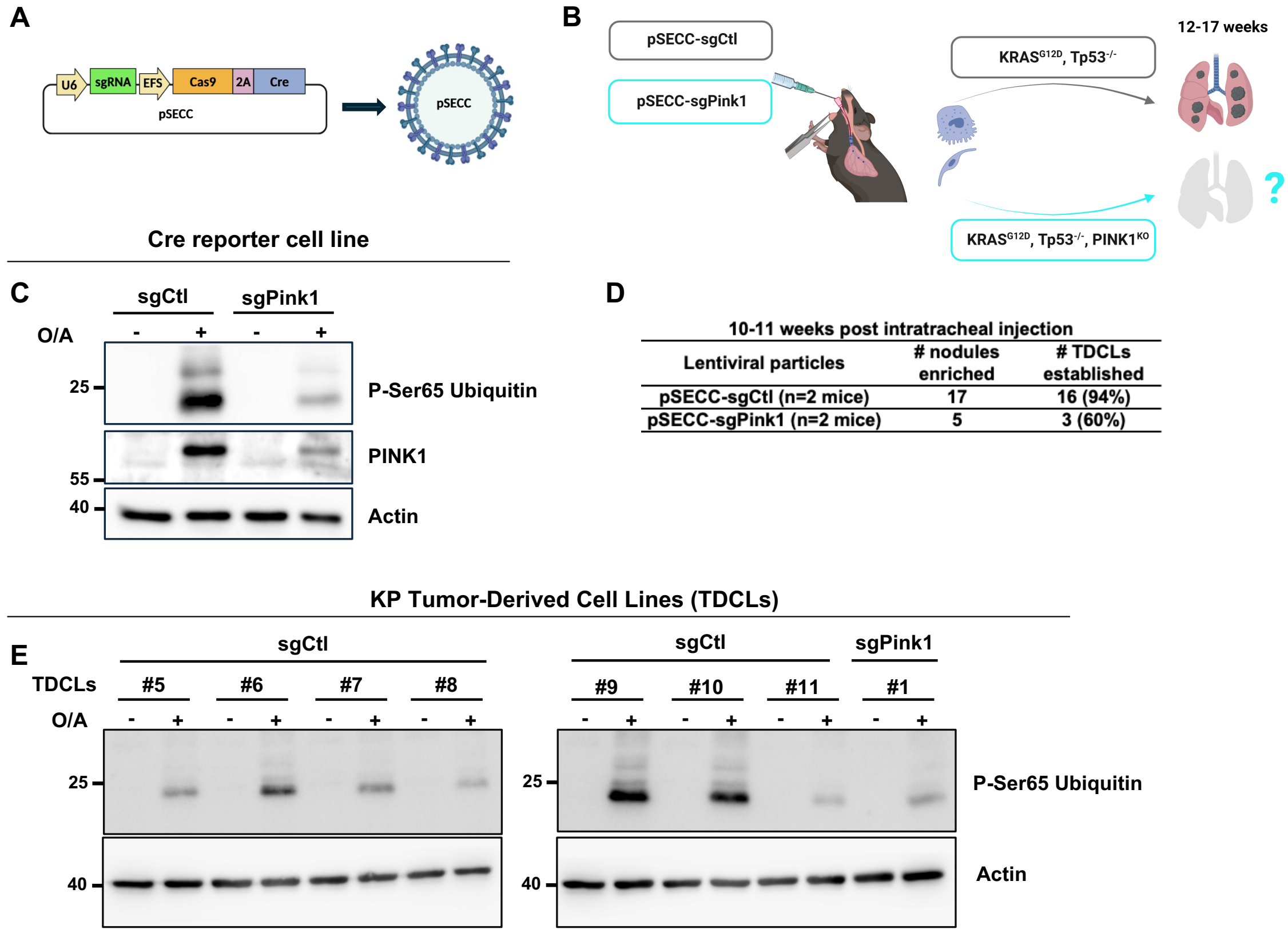

Fig. S4. Pink1 deletion inhibits lung tumor progression in mice by affecting cell transformation.

(A) pSECC bi-cistronic vector enabling simultaneous expression of Cas9, Cre recombinase and sgRNA upon infection. Created in BioRender. Gonçalves, D. (2026) https://BioRender.com/w4k15ug**. (B)** Schematic overview of Pink1 deletion in tumor *in vivo*. Lentiviral particles-containing either pSECC-sgCtl or pSECC-sgPink1 are administered intratracheally to KP mice. Tumor burden is evaluated 12-17 weeks post injection. Created in BioRender. Gonçalves, D. (2026) https://BioRender.com/j7kvyzb. **(C)** pSECC lentiviral vector validation upon GGC9 reporter cell line infection. High GFP sorted GGC9 are treated 24h with either vehicle or oligomycin and antimycin A (5µM and 4µM respectively). **(D)** 10-11 weeks post-intratracheal injection of lentiviral particles containing either pSECC-sgCtl or pSECC-sgPink1, TDCLs were established from isolated nodules. 2 pSECC-sgCtl and 2 pSECC-sgPink1 mice were used. 17 nodules were isolated from pSECC-sgCtl mice (10 for #1 and 7 for #2) and 5 from pSECC-sgPink1 mice (4 for #1 and 1 for #2). 16 TDLCs out of 17 (96%) were established from KPsgCtl nodules while 3 TDLCs out of 5 (60%) were from KPsgPink1 nodules. **(E)** Western blot of TDCLs KPsgCtl and KPsgPink1 treated in vitro with either vehicle or oligomycin and antimycin A (1µM each) for 24h**.**

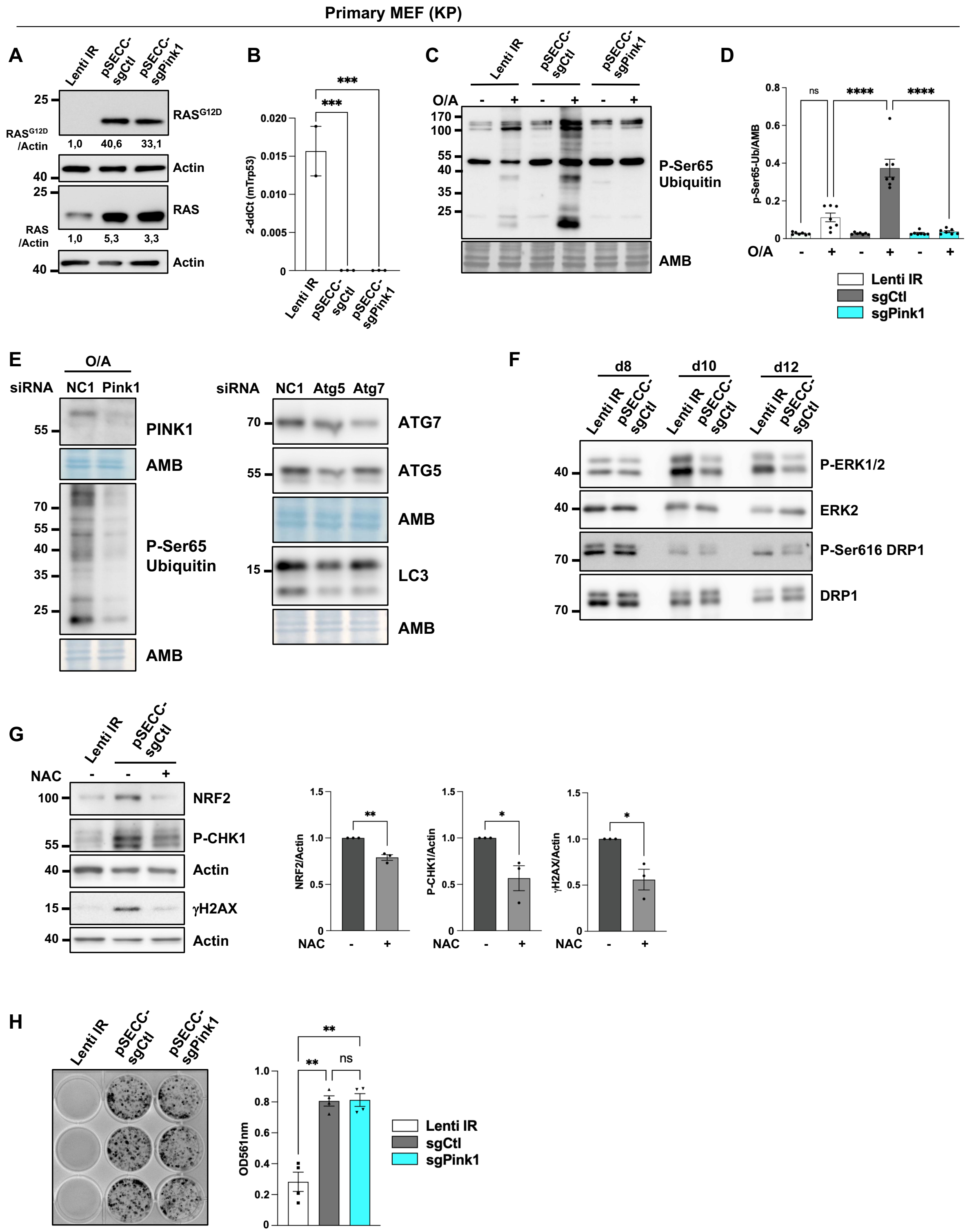

Fig. S5. Oncogenic KRasG12D and p53 deletion promote PINK1-dependent mitophagy that supports cellular transformation.

(A) Analysis of Ras expression (G12D specific and total) in total protein extract from pMEF infected with irrelevant (named lenti IR), pSECC-sgCtl or pSECC-sgPink1 lentiviral particles. **(B)**

SYBR Green-based RTqPCR analysis of Trp53 expression in transduced pMEF as in (F). Data are represented as mean ± SEM, 2-3 independent experiments. One-way ANOVA, ***adj Pval= 0.0008. **(C)** Western blot of pMEFs from KP-QC mice transduced with irrelevant, pSECC-sgCtl or pSECC-sgPink1 lentiviral particles and treated with either vehicle or oligomycin and antimycin A (1µM each) for 24h. AMB: amidoBlack. **(D)** Corresponding semi-quantification of P-Ser65 Ubiquitin signal, normalized to amidoblack, using Image J software. Data are represented as mean ± SEM, 7 independent experiments. 2-way ANOVA, ****P<0.0001. **(E)** Assessment of Pink1, Atg5 and Atg7 knockdown by siRNA transfection in pMEFs, thereafter transduced with pSECC-sgCtl lentiviral particles and tested in 2D clonogenic assay. For Pink1 knockdown, pMEFs were treated 24h with oligomycin and antimycin A (1µM each). **(F)** P-ERK1/2, ERK2, P-Ser616 DRP1 and DRP1 protein expression by Western blot in pMEFs 8, 10 and 12 days post transduction with either irrelevant or pSECC lentiviral particles. **(G)** NRF2, P-CHK1, γH2AX western blot and corresponding semi quantification from pMEFs transduced with irrelevant or pSECC-sgCtl lentiviral particles, then treated with 4mM NAC for 3 days. Data are represented as fold expression to pSECC-sgCtl ± SEM, n=3 independent experiments. Student t-test statistical analyzis. **(H)** 2D clonogenic assays and corresponding quantification done on long-term cultured pMEFs transduced with either irrelevant, pSECC-sgCtl or pSECC-sgPink1 lentiviral particles. Data are represented as mean ± SEM, n=3-4 independent experiments. Brown Forsythe and Welch ANOVA, ** adj Pval= 0.0019 and 0.0023 (lenti IR vs pSECC-sgCtl and lenti IR vs pSECC-sgPink1 respectively).

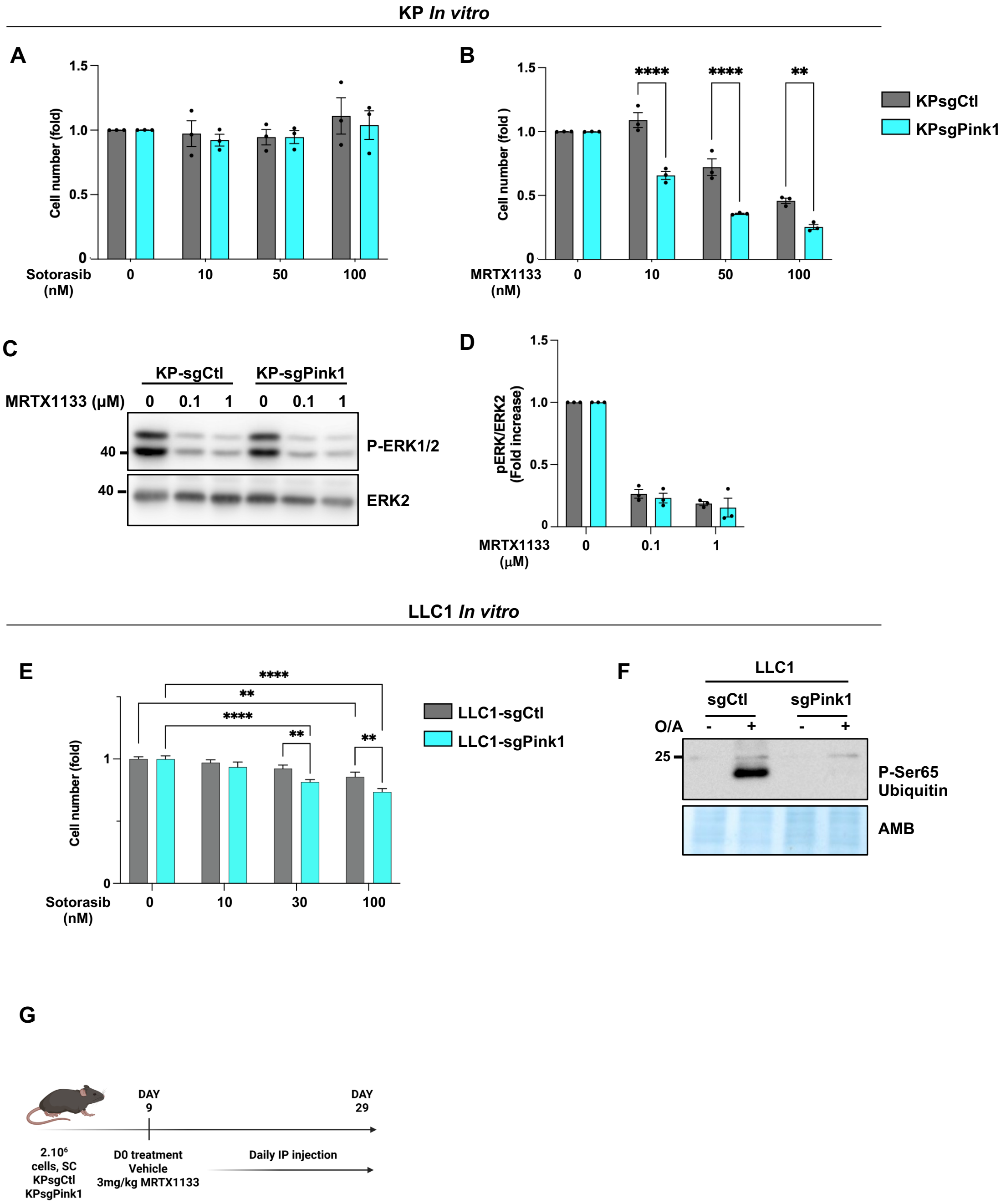

Fig. S6. Pink1 deletion sensitizes Kras mutated cell lines to *oncogenic KRas* inhibition.

(A-B) Sotorasib **(A)** and MRTX1133 **(B)** dose-response in KPsgCtl and KPsgPink1 cell lines. After 72h, DAPI negative cells were counted by flow cytometry. Data are represented as mean fold increase to DMSO ± SEM, n=3 independent experiments. 2-way ANOVA, ****P<0.0001, **P= 0.0027. **(C)** P-ERK1/2 protein expression and quantification in KPsgCtl and KPsgPink1 cell lines upon MRTX1133. Cells were treated for 6h with 0, 0.1 and 1µM of MRTX1133. **(D)** Corresponding semi-quantification. Data are represented as mean fold increase ± SEM, 2-way

ANOVA, ns: non-significant. **(E)** Dose-response of Sotorasib in LLC1 sgCtl and LLC1 sgPink1 cell lines. After 24h, DAPI negative cells are counted by flow cytometry. Data are represented as mean ± SEM, n=3 independent experiments. 2-way ANOVA. **(F)** Assessment of Pink1 activity in LLC1 sgCtl and sgPink1 by Western Blot. Cells were treated for 24h with oligomycin and antimycin A (1µM each). **(G)** *In vivo* experimental design of KPsgCtl and KPsgPink1 allograft tumors treated with 3mg/kg MRTX1133. Created in BioRender. Gonçalves, D. (2026) https://BioRender.com/1ys0scz.
